# Extensive allele mining discovers novel genetic diversity in the loci controlling frost tolerance in barley

**DOI:** 10.1007/s00122-021-03985-x

**Published:** 2021-11-10

**Authors:** Davide Guerra, Caterina Morcia, Franz Badeck, Fulvia Rizza, Stefano Delbono, Enrico Francia, Justyna Anna Milc, Istvan Monostori, Gabor Galiba, Luigi Cattivelli, Alessandro Tondelli

**Affiliations:** 1 Council for Agricultural Research and Economics - Research Centre for Genomics and Bioinformatics, Via S. Protaso 302, 29017 Fiorenzuola d’Arda , PC Italy; 2 Department of Life Sciences, University of Modena and Reggio Emilia, Via Amendola 2, Pad. Besta, 42122 Reggio Emilia, Italy; 3 Centre for Agricultural Research, Agricultural Institute, Eötvös Loránd Research Network, Martonvásár, 2462 Hungary; 4 Department of Environmental Sustainability, Festetics Doctoral School, IES, Hungarian University of Agriculture and Life Sciences, Georgikon Campus, Keszthely, 8360 Hungary

## Abstract

**Key message:**

Exome sequencing-based allele mining for frost tolerance suggests HvCBF14 rather than CNV at Fr-H2 locus is the main responsible of frost tolerance in barley.

**Abstract:**

Wild relatives, landraces and old cultivars of barley represent a reservoir of untapped and potentially important genes for crop improvement, and the recent sequencing technologies provide the opportunity to mine the existing genetic diversity and to identify new genes/alleles for the traits of interest. In the present study, we use frost tolerance and vernalization requirement as case studies to demonstrate the power of allele mining carried out on exome sequencing data generated from > 400 barley accessions. New deletions in the first intron of *VRN-H1* were identified and linked to a reduced vernalization requirement, while the allelic diversity of *HvCBF2a*, *HvCBF4b* and *HvCBF14* was investigated by combining the analysis of SNPs and read counts. This approach has proven very effective to identify gene paralogs and copy number variants of *HvCBF2* and the *HvCBF4b-HvCBF2a* segment. A multiple linear regression model which considers allelic variation at these genes suggests a major involvement of *HvCBF14,* rather than copy number variation of *HvCBF4b-HvCBF2a,* in controlling frost tolerance in barley. Overall, the present study provides powerful resource and tools to discover novel alleles at relevant genes in barley.

**Supplementary Information:**

The online version contains supplementary material available at 10.1007/s00122-021-03985-x.

## Introduction

Cultivated barley (*Hordeum vulgare ssp. vulgare* L.) is the fourth most important cereal crop employed for feed, malt production and, marginally, for preparing food products (Newton et al. 2011). Its origins date back to about 10,000 years ago when it was domesticated in the Fertile Crescent from its wild progenitor *Hordeum vulgare ssp. spontaneum* (C. Koch) Thell. Since then, its cultivation has spread worldwide contributing to the development of mankind by supplying sugars, proteins and fibres (Schmid et al. 2018) and its germplasm has been shaped by the environmental conditions and, in the last century, by plant breeding. Retrospective analysis showed that the genetic progress has been slowing in the last 30 years, a result associated with a reduction of useful alleles circulating in the cultivated material (Dawson et al. 2015). Indeed, modern barley varieties have always been developed through successive rounds of crosses and strong selection to increase yield and product quality. This process finally led to a reduction of the genetic diversity in the cultivated gene pool (Dempewolf et al. 2017). Wild crop relatives, landraces and/or old cultivars held in genebanks, represent a reservoir of untapped and potentially important alleles that can be exploited for further crop improvement (Dawson et al., 2015; Dempewolf et al. 2017) and there is evidence suggesting that searching for new allelic variants in gene bank seed stocks is a powerful strategy to increase the diversity available for plant breeding (Brozynska et al. 2016; Lopes et al. 2015).

The systematic research, identification and characterization of new allelic variants for one or multiple genes in large and diverse collections are referred to as “allele mining”. In barley, the usefulness of allele mining was already tested in several reports dedicated to biotic and abiotic stresses (Yang et al., 2014; Spies et al. 2012; Cseri et al., 2011; Stein et al. 2005; Hofinger et al. 2011). Nevertheless, allele mining studies have mainly targeted only one or few genes at a time. A more genome-wide approach to run an efficient allele mining effort can be achieved using whole genome resequencing data (i.e. in rice the 3,^000^ rice genomes project, 2014). In crops with a very large genome (i.e. barley, Mascher et al. 2017), exome capture and sequencing is a useful alternative, since it reduces genome complexity and sequencing costs by focusing only on the gene space. The exome sequencing approach in barley (Mascher et al. 2013) was first used to investigate the environmental adaptation displayed by a collection of 267 landrace and wild relative (*H. vulgare ssp. spontaneum*) genotypes (Russell et al. 2016) and more recently to elucidate the genetic diversity of a barley collection of 403 accessions including formally bred cultivars, landraces and wild barleys developed in the frame of the European project WHEALBI (Bustos-Kors et al. 2019). This germplasm collection includes accessions from 72 different countries covering the geographic range of barley cultivation. When the exome sequence data generated from the 403 accessions were mapped against the barley reference genome assembly (Mascher et al. 2017), more than 64 million single nucleotide polymorphisms (SNPs) were found (Bustos-Kors et al. 2019). Moreover, in the latter study, the use for the first time of a high-quality reference genome assembly increased the accuracy of variant discovery.

Climate change is predicted to significantly affect the global crop distribution and yield mainly because of the increase of mean winter temperatures (Stocker 2014). This in turn will favour the expansion of barley cultivation towards Northern latitudes (Martin et al. 2017) and will foster the use of facultative and spring cultivars for winter sowing in warmer regions (Muñoz-Amatriaín et al. 2020). In this scenario, spring varieties with some degree of frost resistance could be relevant to avoid damage due to erratic winter frost in southern latitudes and late frost after emergence in northern regions (Tondelli et al. 2014).

Frost tolerance in barley is determined by two major QTL on chromosome 5H (Stockinger 2021; Francia et al. 2004). The first QTL (*Frost resistance-H1*, *Fr-H1*) co-segregates with *VRN-H1*, whose allelic variation in barley is associated with variation in frost tolerance (Cuesta-Marcos et al. 2015; Rizza et al. 2011, 2016) and vernalization requirement (Laurie et al. 1995; von Zitzewitz et al. 2005; Trevaskis et al. 2003, 2007). Plants that carry the wild, recessive *vrn-H1* allele show a prolonged vegetative phase and require exposure to low temperatures to induce flowering (winter growth habit). By contrast, plants that carry the dominant *VRN-H1* alleles, characterized by InDels of different length in the first intron or promoter of the gene (Yan et al. 2003; Hemming et al. 2009), have a shorter vegetative phase and the ability to flower irrespective of low temperatures exposure (a feature of spring cultivars; Trevaskis et al. 2007; Hemming et al. 2009). A third category, known as facultative genotypes, is defined by the presence of the winter *vrn-H1* allele and the complete deletion of a second locus referred to as *VRN-H2* (Takahashi and Yasuda 1971; Rizza et al. 2016; von Zitzewitz et al. 2005). Facultative genotypes show a high degree of frost tolerance and can be sown anytime without delaying plant development (Muñoz-Amatriaín et al. 2020).

The second QTL (*Frost resistance-H2*, *Fr-H2*) co-segregates with a cluster of at least 13 *C-repeat Binding Factors* (*CBF*) (Pasquariello et al. 2014; Stockinger et al. 2007; Francia et al. 2007) characterized by the presence of the highly conserved APETALA2 (AP2) domain that regulates the expression of cold-regulated (COR) genes (Tondelli et al. 2011). Their key role in frost tolerance has been widely proved in several plant species. Wheat and barley plants overexpressing a single *CBF* accumulate COR gene transcripts even in absence of cold stress and are more frost tolerant than their wild type (Morran et al. 2011; Soltész et al. 2013; Jeknić et al. 2014). Allele mining of four *CBFs* in a panel of 216 spring and winter accessions identified two allelic variants of *HvCBF14* associated with frost tolerance (Fricano et al. 2009). Also, there are indications that copy number variation at *Fr-H2* could determine different levels of frost tolerance in barley. Indeed, it has been shown that copy number (CN) of a 17 Kb segment on chromosome 5H harbouring *HvCBF2a* and *HvCBF4b* genes is highly correlated to frost tolerance in barley (Knox et al. 2010; Dhillon et al. 2017; Francia et al. 2016). According to the latter studies, among the 13 *CBFs* at the *Fr-H2* locus the best candidates for frost tolerance are *HvCBF14*, *HvCBF2a* and *HvCBF4b*. The genetic basis of frost tolerance in barley is well described particularly in winter cultivars or in winter versus spring comparison; nevertheless, the increasing interest in spring and facultative cultivars suggests an additional investigation also within spring germplasm to search for novel alleles conferring a wider adaptation to winter conditions. A previous work has found *FR-H1* and *FR-H2* as major loci controlling frost tolerance in spring barley (Tondelli et al. 2014). In this study, we provide evidence on how the whole-exome sequencing (WES) data generated by Bustos-Korts et al. (2019) on a panel of 403 barley accessions organized in the frame of the EU-project WHEALBI (https://www.whealbi.eu/) can be exploited for extensive allele mining. We focused on the genes controlling frost tolerance for which allelic variation, both SNP and structural variation including copy number variation (CNV), has already been identified and linked to frost tolerance and vernalization requirement. In particular, we aimed at (1) identifying novel structural variation at the *VRN-H1*; (2) identifying SNPs and CNV at CBF genes at the Fr-H2 locus; (3) investigating the contribution of molecular variation at *Fr-H2* to frost tolerance in a spring barley genetic background.

## Materials and methods

### Plant materials

The genetic material used in this study has been built in the frame of the EU-project WHEALBI (https://www.whealbi.eu/) and exhaustively described in recent reports (Bustos‐Korts et al. 2019; Bretani et al. 2020). In brief, the collection of 403 barley accessions includes 188 formally-bred cultivars, 185 landraces and 22 wild barleys (*H. spontaneum subsp. spontaneum* and *H. spontaneum subsp. agriocrithon*) and 8 accessions for which the assignment to a category was doubtful (Bustos‐Korts et al. 2019). Overall, the accessions represent a wide geographic diversity being collected in 72 countries covering a wide range of latitudes and longitudes. For landraces and wild barleys, the geographic coordinates of the collection sites are available, while for formally bred cultivars from different countries, the coordinates of the capital city were arbitrarily used.

### Sequences processing, alignment, SNP calling and structural variant discovery

Detailed procedure used for library preparation, sequences processing, alignment of reads and SNP calling have been already reported in previous articles (Bustos-Kors et al. 2019; Bretani et al. 2020). Since the barley reference genome was assembled from the spring cultivar Morex (Mascher et al. 2017) which carries a large deletion in the first intron of *VRN-H1* and a full deletion of the *VRN-H2* locus (Yan et al. 2004; Dubcovsky et al. 2005), we used a customized reference genome for the molecular characterization of the two loci. More in detail, the Morex dominant *VRN-H1* allele was replaced by the recessive allele of the winter cultivar Strider (Genebank: AY750993), while a new pseudomolecule was obtained by concatenating head-to-tail the sequences of the genes at *VRN-H2* (GenBank DQ492699.1, DQ492696.1) and used to map the whole-exome sequencing reads. To detect and genotype InDels larger than 500 bp in the *VRN-H1* gene Delly v0.7.7 (Rausch et al. 2012) with default parameters was employed. The read count was used to call the presence/absence of *VRN-H2* that represents the major source of variation at this locus.

### Copy number detection using read count

The number of reads mapping to the coding region of *HvCBF2a* and *HvCBF4b* was obtained for all genotypes by using BCFtools version 1.4.1. The read count was then normalized by the length of the captured region (data available on https://sequencing.roche.com website in 120426_Barley_BEC_D04_primary_targets.bed) and by the total mapped reads from the WES experiment. Finally, the copy number of both genes was expressed as the ratio between the normalized read count of each genotype and Morex that is known to harbour a single copy of both *HvCBF2a* and *HvCBF4b* (Francia et al. 2016).

### Real-time qPCR

For each genotype, leaves from 2-week-old plants were collected and finely ground in liquid Nitrogen. Three hundred milligrams of ground tissue was extracted with Wizard Kit (Promega Corporation, Madison, Wisconsin, USA), and obtained DNAs were measured using Qubit™ fluorometer in combination with the Qubit™ dsDNA BR Assay kit (Invitrogen by Thermo Fisher Scientific).

The primers amplification efficiency was evaluated in Real-Time qPCR using five tenfold dilution series of barley genomic DNA (from 50 to 0.005 ng) in duplicate. Real-time qPCR was carried out in a final volume of 25 μl containing 12.5 μl of GoTaq® qPCR Master Mix 2X (Promega, USA), 0.25 μl of Reference Dye ROX 100X (Promega), 0.3 μl of both the forward and the reverse primer (10 μM), 5 μl of DNA template and 6.65 μl of water. PCR was performed on a 7300 Real-time PCR System (Applied Biosystems) using the following cycling protocol: 95 °C for 10 min, 40 cycles of 95 °C for 15 s and 60 °C for 1 min. To evaluate the specificity of the amplified product, a dissociation curve was included and a negative control without template was run with every assay. Using the direct method of calibration standard dilution curve and slope calculation, real-time qPCR efficiency (E) was determined as *E* = 10( − 1/slope) − 1.

### Copy number validation by digital PCR

Digital PCR was performed using QuantStudioTM 3D Digital PCR System (Applied Biosystems by Life Technologies). The reaction mixture was prepared in a final volume of 16 µl containing 8 µl of QuantStudioTM 3D Digital PCR 2X Master Mix, 0.72 µl of each primer at 20 µM, 0.32 µl of FAM and VIC-MGB probes at 10 µM, 2 µl of DNA (4 ng/µl) and nuclease free-water. Also, a negative control with nuclease-free water as template was added. Fifteen microliters of reaction mixture was loaded onto the QuantStudioTM 3D Digital PCR chips using QuantStudioTM 3D Digital chip loader, according to manufacturer protocol. Amplifications were performed in ProFlexTM 2Xflat PCR System Thermocycler (Applied Biosystems by Life Technologies) under the following conditions: 96 °C for 10 min, 45 cycle of 60 °C annealing for 2 min and 98 °C denaturation for 30 s, followed by 60 °C for 2 min and 10 °C. End-point fluorescence data were collected in QuantStudioTM 3D Digital PCR Instrument, and files generated were analysed using cloud-based platform QuantStudioTM 3D AnalysisSuite digital PCR software, version 3.1.6. The software yields absolute Copies/µL and Target/Total results.

### Analysis of nucleotide diversity

Complete nucleotide sequences of all the genes used for this study were obtained for all genotypes from variant file using BCFtools consensus (version 1.4.1) command that apply variants found in vcf file to a reference sequence. Multiple alignments were generated with ClustalW and diversity statistics (nucleotide diversity index, Watterson’s estimator of nucleotide diversity, number of different haplotypes, haplotype diversity) and haplotypes were defined by DNASP 5.10.01.

### Vernalization requirement experiment

To determine the vernalization requirement of the different *VRN-H1* alleles, 15 barley genotypes representing 9 alleles were used. All genotypes carry the wild dominant allele at the *VRN-H2* locus. For each genotype 5 seeds were vernalized at 4 °C for 0, 2, 4 and 6 weeks under dark conditions in petri dishes containing wet paper offset through weeks to allow plants to terminate vernalization simultaneously. At the end of the 6th week, seedlings were sown in pots filled with turf and moved to a growth chamber under long day conditions (16 h light, 8 h dark) at 22/19 °C. To move all the plants to the growth chamber the same day, the sowing of seeds for each vernalization treatment was carried out in a scalar manner. The number of days required for the ear to emerge from the sheath of the main shoot was counted from sowing. Finally, five plants per genotype were measured.

### Frost tolerance assay

To assess frost resistance, 10 individuals from each genotype were assayed in 5 replications. Seeds were germinated in Petri dishes for 1 day at 25 °C and 3 days at 4 °C and then planted into wooden boxes containing a 2:1:1 (v/v/v) mixture of soil, sand and humus. Each wooden box contains 150 plants (13 genotypes plus 2 control lines; 10 plants/genotype) planted in a randomized order, and the wooden boxes were further randomized in a single growth chamber and rearranged regularly during the whole experiment. Plantlets were grown in a regime of decreasing temperature and illumination in plant growth chamber till the four-leaf stage (about 4–5 weeks). During this period, the soil moisture content was monitored daily in each box, by measuring the conductivity of the soil using a conductivity meter Type OK-102/1 (Radelkis) and adjusted to 120–170 µS. Plants were then further exposed to a hardening temperature of 3/0 °C (day/night) for 1 week without irrigation. Subsequently, the frost treatment was imposed for 24 h in the dark at − 6 and − 9 °C. After the frost treatment, the temperature was raised by 2 °C/h steps to + 1 °C, and the plants were kept at this temperature for 24 h. At this point, leaves were cut 1 cm above soil level and the boxes transferred at 17/16 °C (day/night) with 14 h/day illumination for 3 weeks. Frost tolerance was estimated after one week and 3 weeks of recovery by assessing the re-growth of the plants, which was scored on a scale running from 0 (death) to 5 (undamaged) as previously described by Vágújfalvi et al. (2003).

For the statistical analysis, the genotype mean survival scores were calculated by fitting a two-way linear model applying routines for sparsely populated matrices with the R-package pheno (Schaber 2007). The mean scores were normally distributed. Multiple linear regression with backward elimination of the genotype mean scores on CNV and alleles of the studied genes was done with R.

### Expression analysis of *HvCBF2a* and *HvCBF4b*

RNA for gene expression analysis was isolated from three independent plants of the barley genotypes WB-247, WB-268, WB-270 and WB-273, to verify the correlation between the number of copies of *HvCBF2a* and *HvCBF4b* and their expression level. The genotypes Morex and Pamina were added as reference spring and facultative barleys carrying one and multiple copies of the *HvCBF2a-HvCBF4b* segment, respectively. Barley seeds were sown in polystyrene seedling tray containing turf and grown in a growth chamber for 7 *d* (first leaf stage) in a daily regime of 8 h light (200 μmol m − 2 s − 1) at 22 °C/19 °C and cold treated at 3 °C in a growth chamber with 300 μmol m − 2 s − 1 for 0, 2 or 4 h. First leaves were sampled at control conditions and after 2 and 4 h of cold treatment from three plants for each genotype and immediately frozen in liquid nitrogen. Tissues were stored at − 80 °C until the use. RNA was extracted using TRI Reagent® following the manufacturer extraction. qRT-PCR analyses were performed using the 7300 Real-Time PCR System (Applied Biosystems). Specific primers were designed using Primer Express™, and GAPDH and β-Actin were used for housekeeping. The relative fold change of the expression levels was obtained by the 2–DDCt (cycle threshold) method (Livak and Schmittgen 2001).

## Results

### Nucleotide diversity at *VRN-H1*

**Fig. 1:**
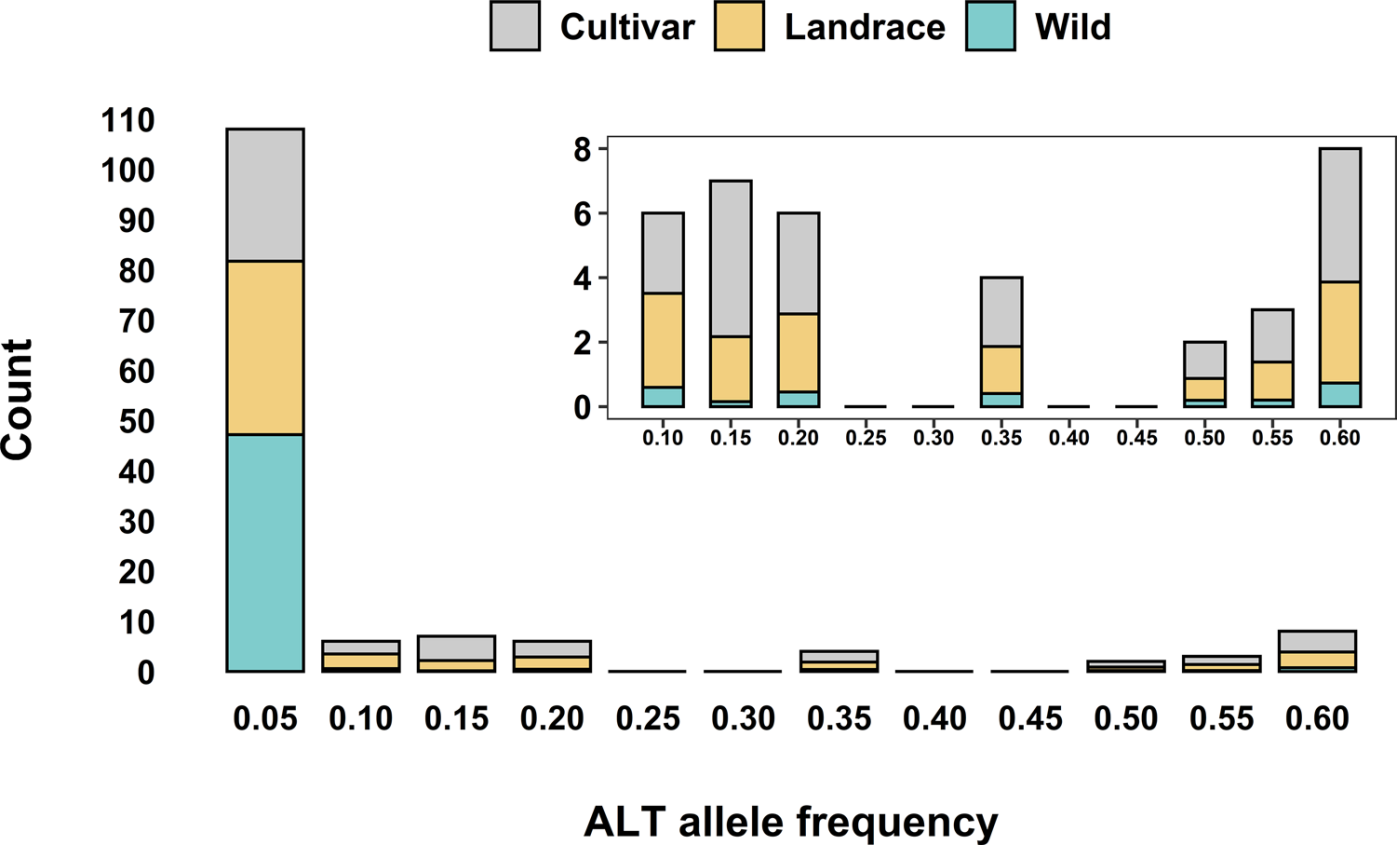
Site frequency spectrum of the alternative allele in the collection. The *X* axis reports the frequency of the alternative allele; the *Y* axis reports the number of SNPs in each bin. Each bar is coloured in relation to the proportion of genotypes, in each frequency class, that belong to one of the three categories: cultivar, landrace and wild. The inset is a magnification of bars from frequency class 0.1–0.6

To mine the natural diversity in the major genes implicated in barley frost tolerance, we used the genomic resource described in Bustos-Korts et al. (2019). The co-segregation between *Fr-H1* and *VRN-H1* highlights the role of growth habit in frost tolerance (Stockinger et al. 2007; Akar et al. 2009; Visioni et al. 2013; Zhu et al. 2014) and motivated the exploitation of the allelic diversity detected in the *VRN-H1* sequence. In the present work, a total of 143 SNPs, 103 transitions (TS) and 40 transversions (TV), have been identified (Table S2)*.* Two and one SNPs are in the first and second exon, respectively, 32 SNPs in regions upstream or downstream to the gene and 108 SNPs in intronic regions. The SNPs in the coding regions represent two synonymous and one missense mutation, respectively, the latter producing a Leucine to Isoleucine change at position 80 of the protein. Most of the SNPs are singletons (SNPs found only in one accession) or very rare in the collection; indeed, 75.4% of them have a frequency below 0.05. Wild genotypes represent the major source of rare alleles contributing 36 out of 47 singletons, followed by landraces and cultivars with 7 and 4 singletons, respectively (Fig. 1). In general, polymorphism is the highest in wild material followed by cultivars and then landraces, possibly because of breeding that aimed at maintaining a higher diversity degree at this locus to better respond to regional needs in terms of vernalization requirements. Indeed, the cultivars in the collection span a wider range of latitude and longitude than landraces (Supplementary Table S1). Pairwise nucleotide diversity (*π*) and Watterson’s nucleotide diversity estimator (*θ*) were also lower in landraces than cultivars (Supplementary Table S3). After pruning all the variants with one or more missing data across the 403 accessions sequenced, 36 SNPs have been retained that form 25 haplotypes (Supplementary Fig. S1), of which 15 are unique in the collection and mainly found in wild barleys. A detailed evaluation of SNP variation and growth habit has ruled out any association between specific haplotypes and barley growth habit suggesting that the natural nucleotide diversity identified in the present study in *VRN-H1* has no impact on it its role in developmental transition.

### Identification of deletions in the first intron of *VRN-H1*

**Fig. 2:**
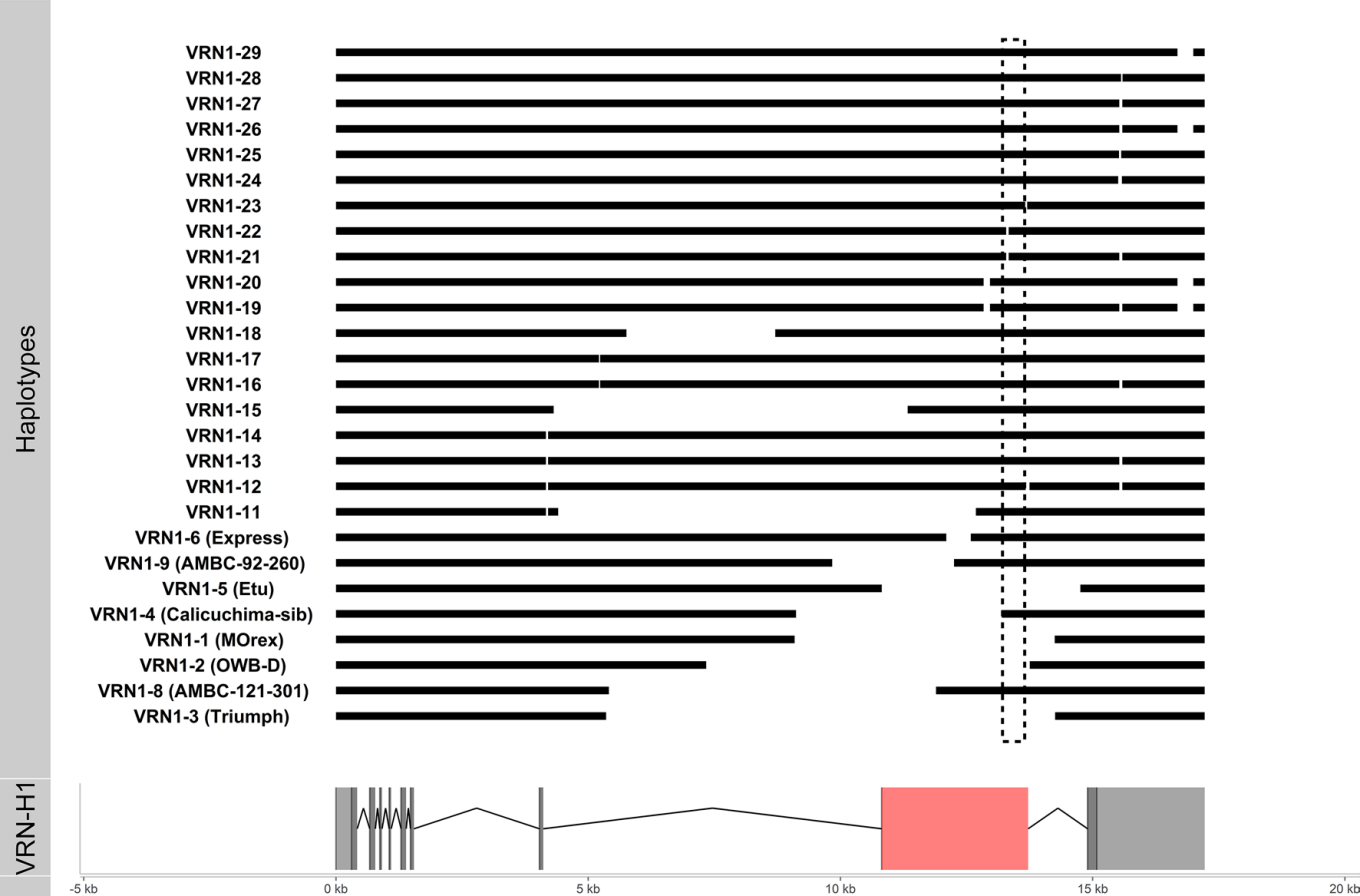
Haplotypes at the VRN-H1 as defined by deletions relative to the cv. Strider allele. The haplotypes are named according to the previous nomenclature in Hemming et al. (2009). Light grey rectangles are UTR regions; dark grey rectangles are exons; introns are represented by the thin black line. The light red and the dashed rectangles indicate the 2.8 Kb regulatory region and the core regulatory region, respectively (Hemming et al. 2009)

As the major source of variation affecting growth habit in barley is linked to large deletions in the first intron of *VRN-H1* (Hemming et al., 2009), we used the exome data to search for intron deletions in the WHEALBI collection. To accomplish this task, the whole-exome sequencing reads were mapped to a customized version of the reference genome since it was assembled from the spring cultivar Morex (Mascher et al. 2017) that carries a deletion of 5.1 Kb in the first intron of *VRN-H1* (Hemming et al., 2009) hindering the identification of structural variants in that region. Accordingly, the Morex allele was replaced by the recessive allele of the winter cultivar Strider (Genebank: AY750993) that represents the wild *VRN-H1* allele. Overall, thirty-one deletions have been identified in the WHEALBI panel (Supplementary Table S4), eight of them already characterized in previous works (Fu et al. 2005; von Zitzewitz et al. 2005; Cockram et al. 2007; Szucs et al. 2007; Hemming et al. 2009). The size of the deletions ranges from 25 to 8904 bp. Twenty-six deletions are in the first intron, of which 13 are in the 2.8 kb regulatory region previously defined (Hemming et al. 2009), while 5 are in the promoter region (Fig. 2). A deletion of 314 bp in the promoter falls between two CarG box motifs, cis elements known to play a key role in the regulation of the wheat *VRN1* (Yan et al., 2003). Overall, the deletions identified 27 different haplotypes (Fig. 2) that we have named following the previous nomenclature proposed by Hemming et al. (2009). The most frequent haplotypes are *VRN1-4*, *VRN1-1*, and the wild recessive allele *VRN1* with 72, 63 and 62 genotypes, respectively. Three large new deletions of 8281, 7019 and 2950 bp, named *VRN1-11*, *VRN1-15,* and *VRN1-18*, have been detected (Fig. 2). These deletions partially remove the 2.8 kb regulatory region in haplotypes *VRN1-11* and *VRN1-15* but not in haplotype *VRN1-18*. Moreover, two small deletions of 76 and 40 bp (haplotypes *VRN1-12* and *VRN1-23*, respectively) are immediately downstream of the Miniature Inverted-repeat Terminal Element (MITE) inside the critical region and may affect the transcriptional regulation of the gene (Fig. 2). No deletions affecting regulatory regions have been found in wild genotypes. Finally, no insertions have been detected in any of the 403 barley genotypes despite the fact that the collection includes the cultivar Varunda which is known to have an insertion of 692 bp in the first intron (Hemming et al. 2009). We speculated the failure to identify this insertion was due to its absence in the target sequence used for the capture design.

To test the effect of the newly detected alleles of *VRN-H1* on vernalization requirement, 15 barley genotypes representing 9 different alleles including Strider and Morex, a winter and a spring genotype, respectively, were vernalized for 0, 2, 4 or 6 weeks and then grown in long day conditions until head emergence. As the growth habit is also dependent on the allelic state at *VRN-H2*, all the genotypes used in this experiment have been selected as carrying the dominant wild allele at this locus. The allelic state at *VRN-H2* was tested using normalized read count to call the presence/absence of the locus, as indicated by previous studies (Yan et al. 2004; Dubcovsky et al. 2005). For the alleles *VRN1-15*, *VRN1-20* and *VRN1-26*, a single representative genotype was available in the whole collection, while for the other four alleles (*VRN1-11*, *VRN1-12*, *VRN1-19*, *VRN1-29*), three or four different genotypes were tested.

**Fig. 3:**
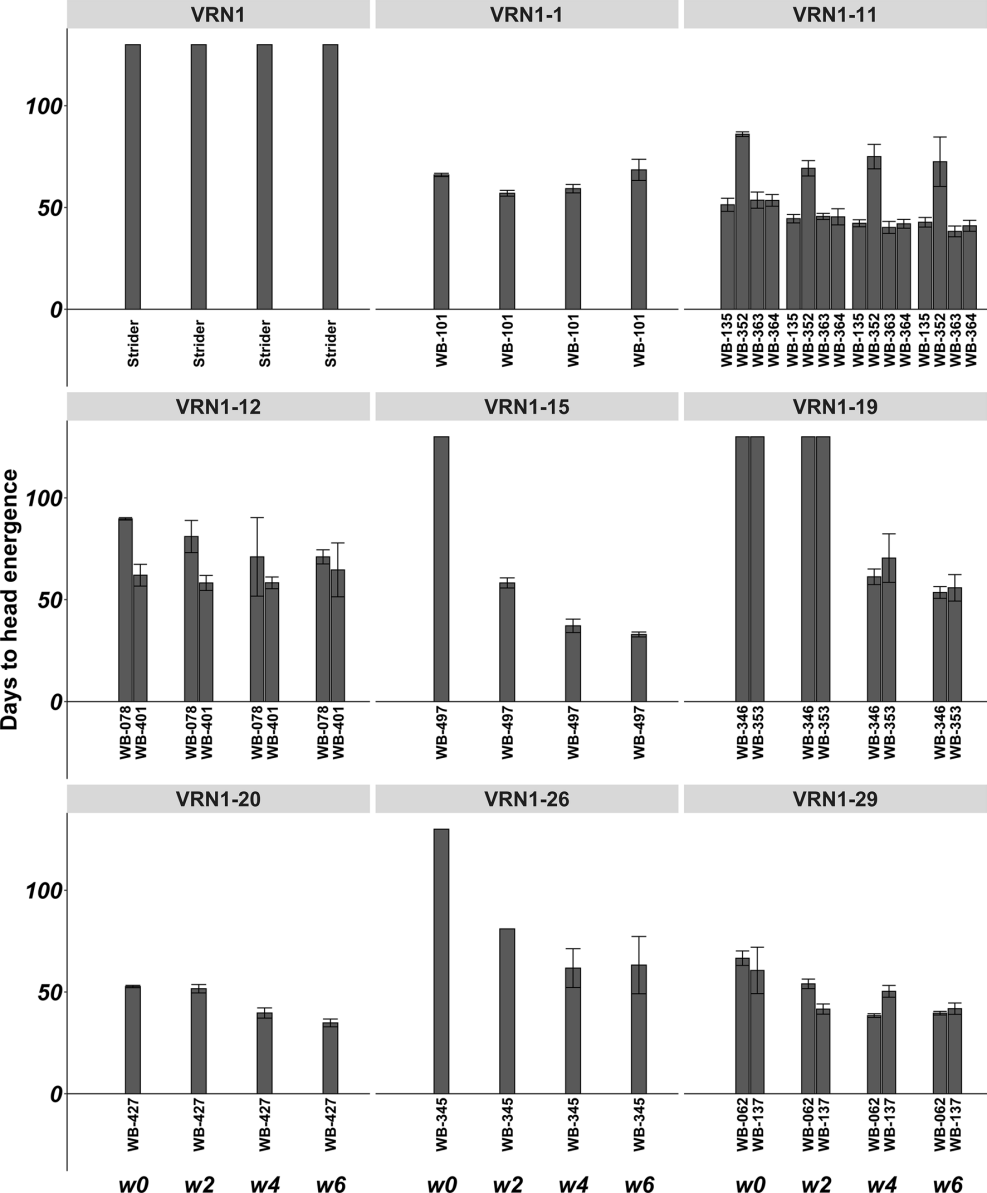
Vernalization response measured as days to head emergence in long days conditions for barley lines carrying different *VRNH1* alleles. Values above 100 indicate no flowering. The *VRN-H1* allele is reported on the top of each panel and, for each allele, the genotype code is reported on *x* axis. The length of vernalization treatment is reported on the bottom of the picture from left to right (0, 2, 4, 6 weeks). For significance of differences between treatment, see ANOVA tables and Tukey test in Supplementary Data S1

All barley genotypes that carry alleles *VRN1-11, VRN1-12, VRN1-20,* and *VRN1-29* can induce flowering without vernalization requirement as the allele *VRN1-1* carried by Morex, the WHEALBY Barley (WB) accession n.101 (WB-101). The vernalization treatment can accelerate the time to flowering in accessions WB-135, WB-363, WB-364 (VRN1-11), WB-427 (VRN1-20), WB-062 and WB-137 (VRN1-29), while WB-352 (VRN1-11) is totally insensitive to vernalization even if it flowers later than the others (Fig. 2). Genotypes WB-078 and WB-401, carrying allele VRN1-12, show a wide variation in flowering time after 4 or 6 weeks of vernalization, respectively, suggesting that this period of vernalization may be critical for accelerating flowering induction (Fig. 2). WB-497 and WB-345 carrying allele *VRN1-15* and *VRN1-26*, respectively, flower only after at least two weeks of vernalization, although longer vernalization treatment strongly accelerate flowering (Fig. 3). Genotypes WB-346 and WB-353, carrying allele *VRN1-19*, flower only after at least 4 weeks of vernalization suggesting a stricter winter growth habit but not as much as Strider.

### Nucleotide diversity at *HvCBF2a*

As several studies suggested *HvCBF2a* as the best candidate at *Fr-H2* locus for frost tolerance (Xue, 2003; Jecknić et al. 2014), we investigated its natural variation in the WHEALBI collection. After removing 59 genotypes with one or more missing data, 9 SNPs and 344 genotypes were retained. All the SNPs are in the coding region outside the conserved AP2 domain (Supplementary Table S5). Five SNPs are missense mutations, while four are synonymous mutations. Interestingly, six SNPs showed a heterozygous state in 52 genotypes (Supplementary Table S5) and the allele balance (the ratio between sequencing reads for the alternative and reference alleles) for the heterozygous SNPs is close to 1 in most of the cases, suggesting that each allele is equally represented in terms of number of reads (data not shown) hence supporting a real heterozygous state. Based on the latter observation, we analysed the 52 genotypes with heterozygous SNPs separately (see next paragraph). For the remaining 292 genotypes, 6 SNPs defined seven different haplotypes (Supplementary Fig. S2), with the most abundant one (haplotype 3) being observed in 104 accessions and corresponding to the Morex *HvCBF2a* allele. Haplotype 1 counts 54 accessions and identifies the *HvCBF2a/2b* gene fusion typical of the cultivar Tremois (Knox et al. 2010). Haplotypes 2 and 4 originate from haplotype 3 and 1, respectively, following a single mutation. Although each haplotype is shared evenly among the WHEALBI accessions in terms of growth habit, biological status, and geographic origin, we observed that haplotype 1 is predominantly present in spring genotypes from the Mediterranean basin (Supplementary Fig. S2). Despite a central role in cold tolerance suggested for *HvCBF2a* (Xue, 2003; Jecknić et al. 2014), no variants predicted to affect protein structure or function have been found in the coding sequence of *HvCBF2a* in the WHEALBI collection.

### Simultaneous presence of *HvCBF2* paralogs is revealed by heterozygous SNPs

As reported above, 52 genotypes are characterized by six heterozygous SNPs at *HvCBF2* (Supplementary Table S5). In a previous work, a similar behaviour has been described in the exome sequence data of three BC_1_F_4_ barley lines for NBS-LRR genes and the authors concluded this behaviour might suggest the presence of gene paralogs in the captured genotypes but not in the reference genome (Cantalapiedra et al. 2016). Since paralogs for barley *HvCBF2* have been already reported (Knox et al. 2010), most likely because of duplication and subsequent differentiation due to mutations, we investigated the heterozygous calls considering them as predictive of such source of variation. Indeed, the reads producing the heterozygous variants can be grouped into two different populations representative of each allele in a proportion that excludes sequencing errors. Furthermore, the heterozygous genomic positions coincide with the polymorphic positions between the known *HvCBF2* paralogs (Supplementary Fig. S3).

**Fig. 4:**
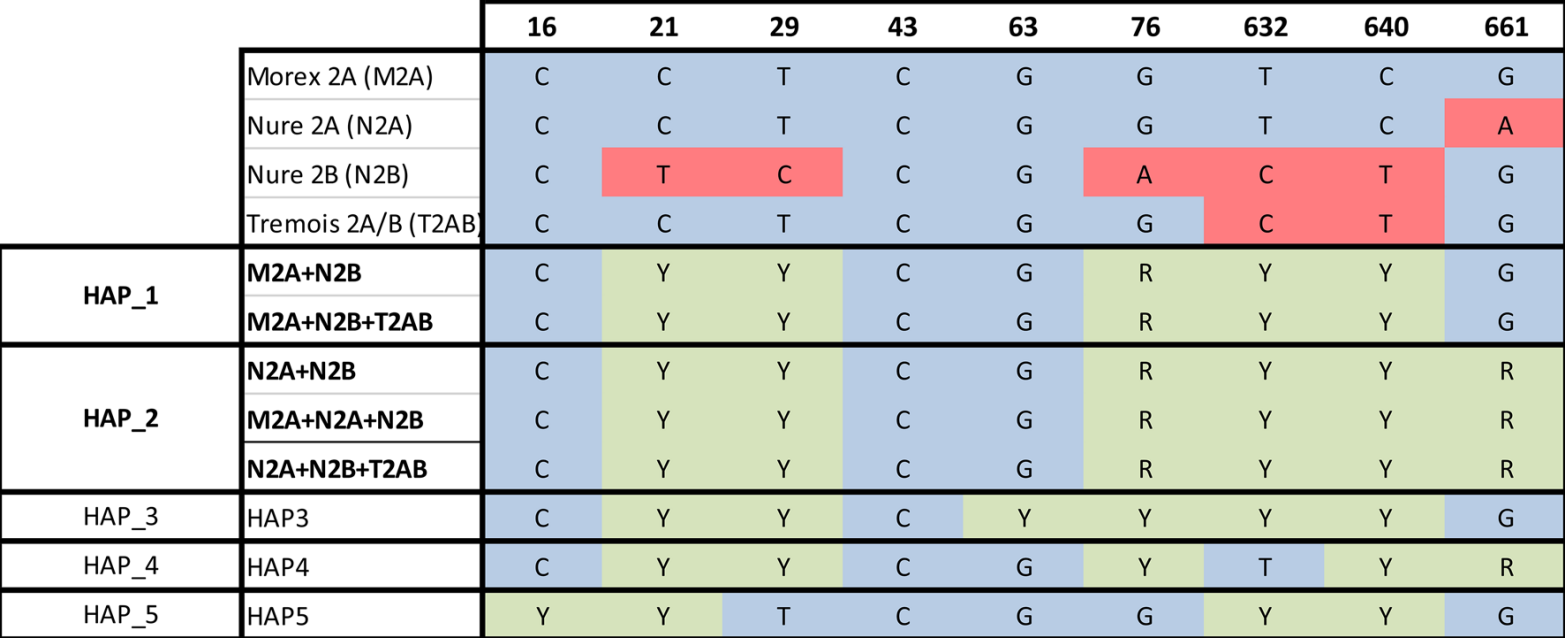
Schematic representation of haplotypes reconstruction for *CBF2* paralogs from variant data. Light blue indicates homozygous reference allele; red indicates the homozygous alternative allele; light violet indicates heterozygous state of the variant position. Heterozygous variants are indicated according to the IUPAC nomenclature (*R* = purines, *Y* = pyrimidines). The first four rows are the haplotypes of known *CBF2* paralogs, while below are the haplotypes inferred in the 36 genotypes with heterozygous calls. The header of each column refers to base pair position of the variant relative to the start codon of *CBF2*

A genotype with two or more paralogs all equally captured by the exome designed probes should provide a higher number of templates in the sequencing reaction. Consequently, once the reads are mapped to a reference with a single copy of the gene, the alignment should highlight a higher read count and can be grouped in 5 different haplotypes likely differing in the combination of the paralogs of *HvCBF2*, while for the remaining 16 genotypes the read count does not support the presence of further paralogs. Two haplotypes are ambiguous since the same haplotype can originate from different paralog combinations (Fig. 4). To validate the haplotypes as inferred from exome data and to solve those that classified as ambiguous, representative accessions for each haplotype were genotyped with a CAPS assay previously developed (Francia et al. 2016). The data generated with the CAPS assay and the paralogs prediction from the exome data confirmed the presence of the already known *HvCBF2* paralogs and allowed the identification of some novel alleles.

Besides the already known *HvCBF2* paralogs (*HvCBF2a*, *HvCBF2b* and *HvCBF2a/b*; Knox et al. 2010), five new haplotypes (Hap_1 to Hap_5 in Fig. 3), representing different combinations of *HvCBF2* paralogs, have been identified by combining heterozygous SNP analysis to read count (Fig. 4). Hap_1, originated from the simultaneous presence of the Nure *HvCBF2a* and *HvCBF2b* alleles is the most frequent haplotype (with 23 genotypes), followed by Hap_2, which arise from the simultaneous presence of the *HvCBF2a* allele of Morex and *HvCBF2b* of Nure. Hap_3 and Hap_4 can be considered variations of Hap_1 and Hap_2 following additional SNP at position 63 and 632, respectively. Finally, a more complex picture concerns Hap_5 which can be originated from the combination of *HvCBF2a/b* and an allele of *HvCBF2a* (Fig. 4).

### Copy number variation at the *HvCBF2a-CBF4b* segment

**Fig. 5:**
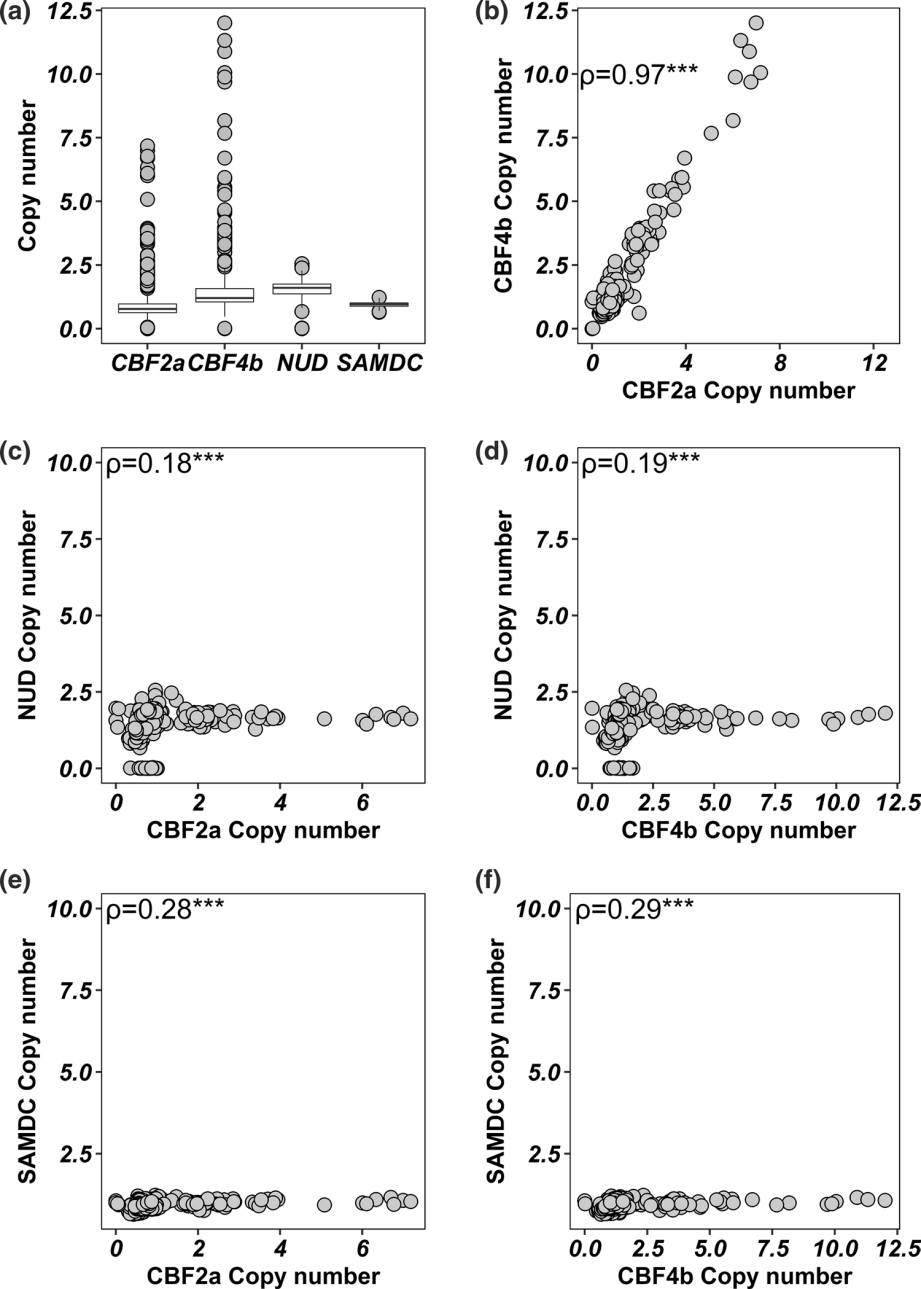
**a** Distribution of copy number (rpkm ratio) for *CBF2a*, *CBF4b,* *NUD and SAMDC*; **b** correlation between copy number of *CBF4b* and *CBF2a*; **c**–**d** correlation between copy number of *NUD* and *CBF2a and CBF4b*; **e**–**f** correlation between copy number of *SAMDC* and *CBF2a* and *CBF4b*

**Fig. 6:**
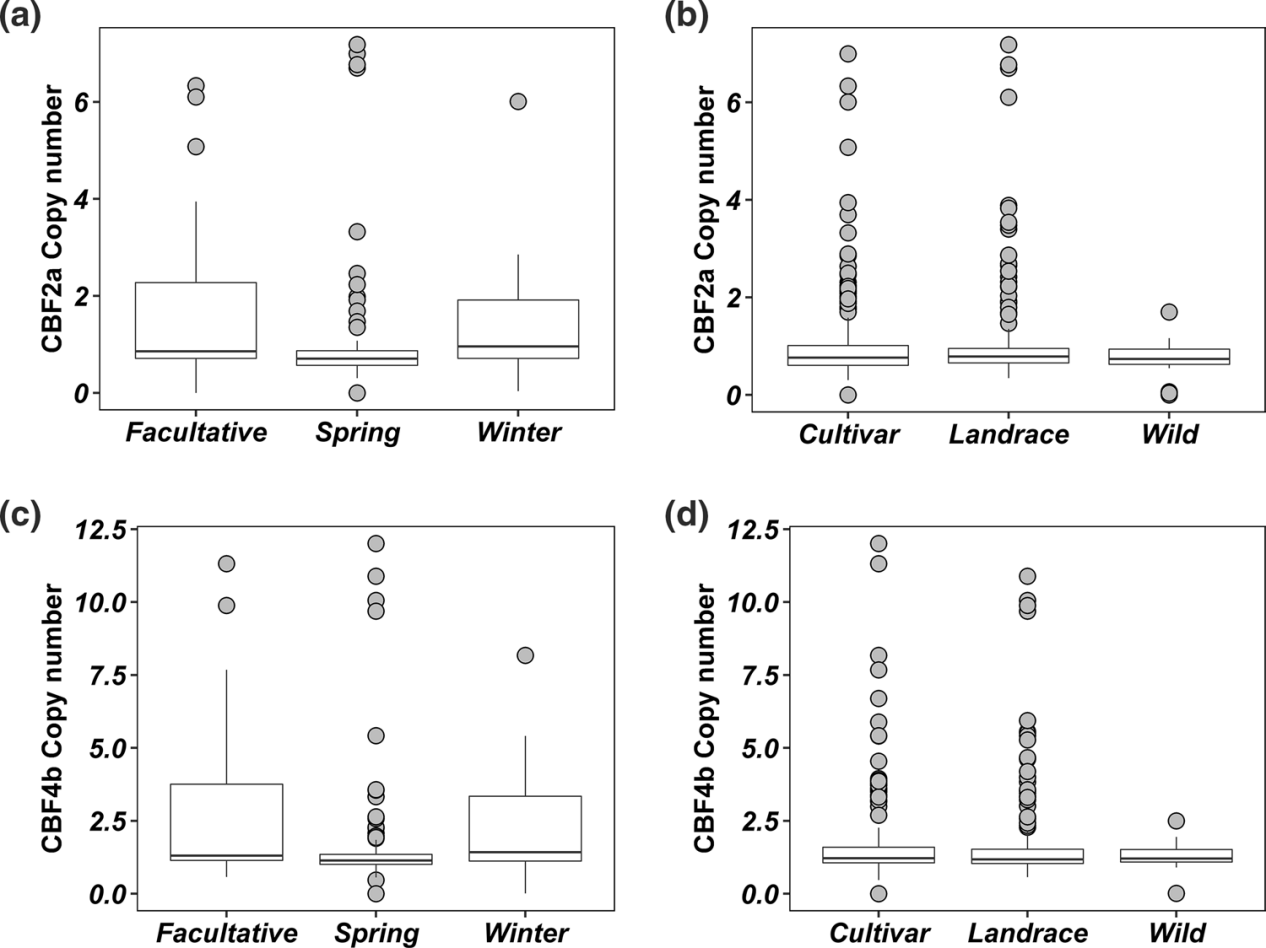
Distribution of copy number variation of *CBF2a* (**a**, **b**) and *CBF4b* (**c**, **d**) among different growth habit (**a**, **c**) and biological status (**b**, **d**) of barley genotypes in the WHEALBI collection

As copy number variation contributes to a further level of diversity at the *Fr-H2* locus and was suggested to be responsible for most of the phenotypic variation associated with frost tolerance (Francia et al. 2016), we explored this additional level of diversity in the WHEALBI collection using the exome data through the analysis of the read count at the *HvCBF2a-HvCBF4b* genomic segment. Raw read count and rpkm (reads per kilobase of target per million mapped reads) of both genes show a large variation in the WHEALBI collection reaching in some cases a very high value (Supplementary Figs. S4 and S5). Next, to get an estimate of the exact copy number of both genes the rpkm has been normalized against the reference cv. Morex that is known to have a single copy of both *HvCBF2a* and *HvCBF4b* (Francia et al. 2016). As an internal control we used the *HvNUD* and *HvSAMDC* genes, which are known to be present as a single copy gene in barley as previously reported (Taketa et al. 2008; Francia et al. 2016). The rpkm ratio confirmed that *HvCBF4b* and *HvCBF2a* show a large diversity in CN in the collection with the highest values reaching 12.00 and 7.2, respectively; the *HvNUD* and *HvSAMDC* genes instead show very little variation with a maximum value of 2.5 and 1.2, respectively (Fig. 5a). The correlation between CN of *HvCBF2a* and *HvCBF4b* is 0.97, while the correlation between *HvNUD* and *HvCBF2a* or *HvCBF4b* is 0.18 and 0.19, respectively, and a similar behaviour can be observed when compared to *HvSAMDC* (Fig. 5b–f). Overall, 46 out of 403 genotypes show a copy number of the *HvCBF2a—HvCBF4b* segment equal or higher than two copies. For both genes, the median of CN and the interquartile range are higher in winter and facultative genotypes than springs, indicating that the former are enriched in genotypes with a high *HvCBF2a*-*HvCBF4b* copy number, although the highest copy number of both genes has been found in spring genotypes (Fig. 6a, c). All genotypes carrying more than two copies of *HvCBF2a*-*HvCBF4b* are cultivars and landraces except one. Indeed, only one *H. spontaneum* accession carries two copies, while all other wild accessions have a single copy of the genes. This finding strongly suggests that the duplication at the *HvCBF2a*-*HvCBF4b* locus may have originated after domestication (Fig. 6b, d). Copy numbers of *HvCBF2a* and *HvCBF4b* were also validated using digital PCR, resulting in a Pearson correlation of 0.96. The CN values obtained from WES or digital PCR are in most cases identical, differing from each other in three out of eleven genotypes (Supplementary Figure S6). Among the different *HvCBF2* paralogs described above, only the Morex-like paralog is affected by CNV (Supplementary Fig. S7).

**Fig. 7:**
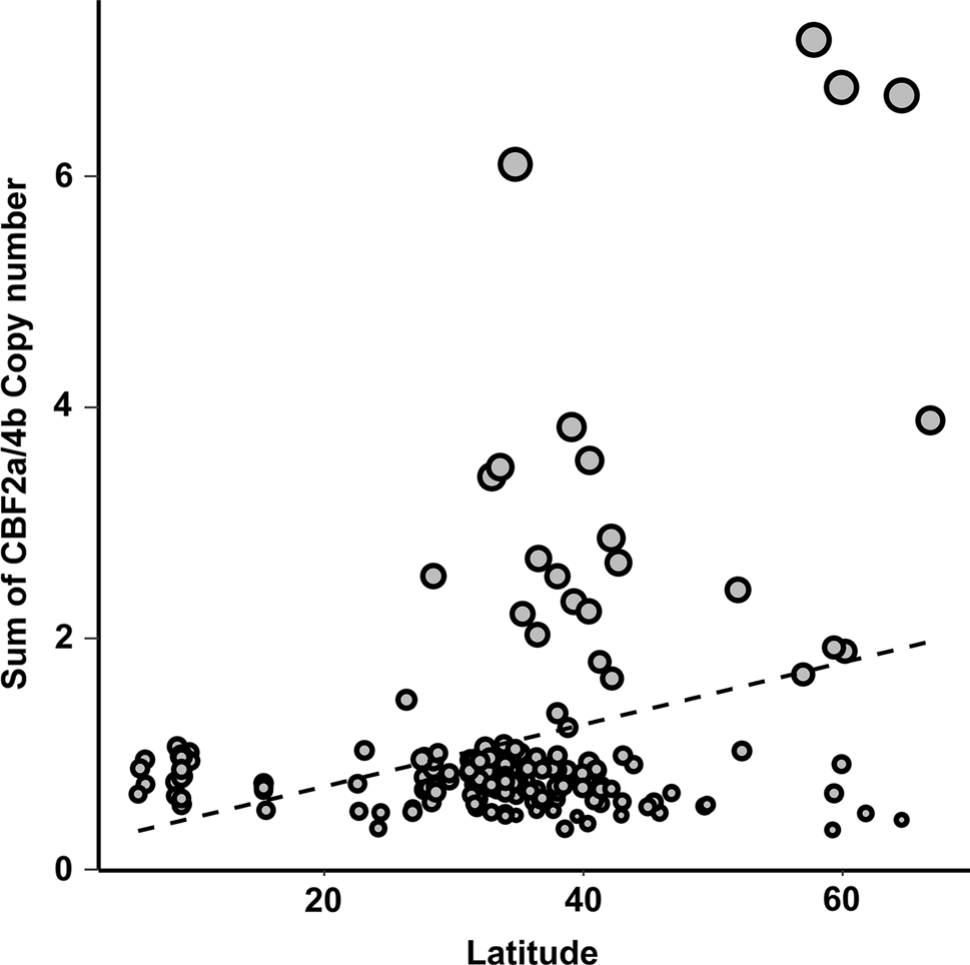
Scatterplot showing the distribution of the sum of copy number of *CBF2a* plus *CBF4b* by latitude of sampling. Size of circles is proportional to the sum of *CBF2a* and *CBF4b* copy number

A geo-reference analysis of the accessions showing CNV showed that most accessions with CNV higher than two were sampled between 30° and 45° of north latitude, the genotypes with the highest value have been sampled above 55° of north latitude, while no genotypes showing CNV are from below 25° (Fig. 7). These findings suggest a role of CNV at the *CBF* locus in the adaptation to environment.

### Nucleotide diversity at HvCBF14 gene

Besides *HvCBF2a*-*HvCBF4b*, *HvCBF14* was also previously suggested as a potential player in frost tolerance in barley (Fricano et al., 2009); therefore, this gene was also investigated in our allele mining approach. A total of 5 SNPs, 1 transition (TS) and 4 transversions (TV), have been identified in the 5-prime end of the gene. One SNP is a synonymous mutation, while the other four are missense mutations. The five SNPs have a frequency of 0.32, 0.004, 0.003, 0.006 and 0.52 and can be grouped in 8 different haplotypes. No significant impact on protein domains could be determined.

### CBFs variants associated with frost tolerance in spring barley

**Fig. 8:**
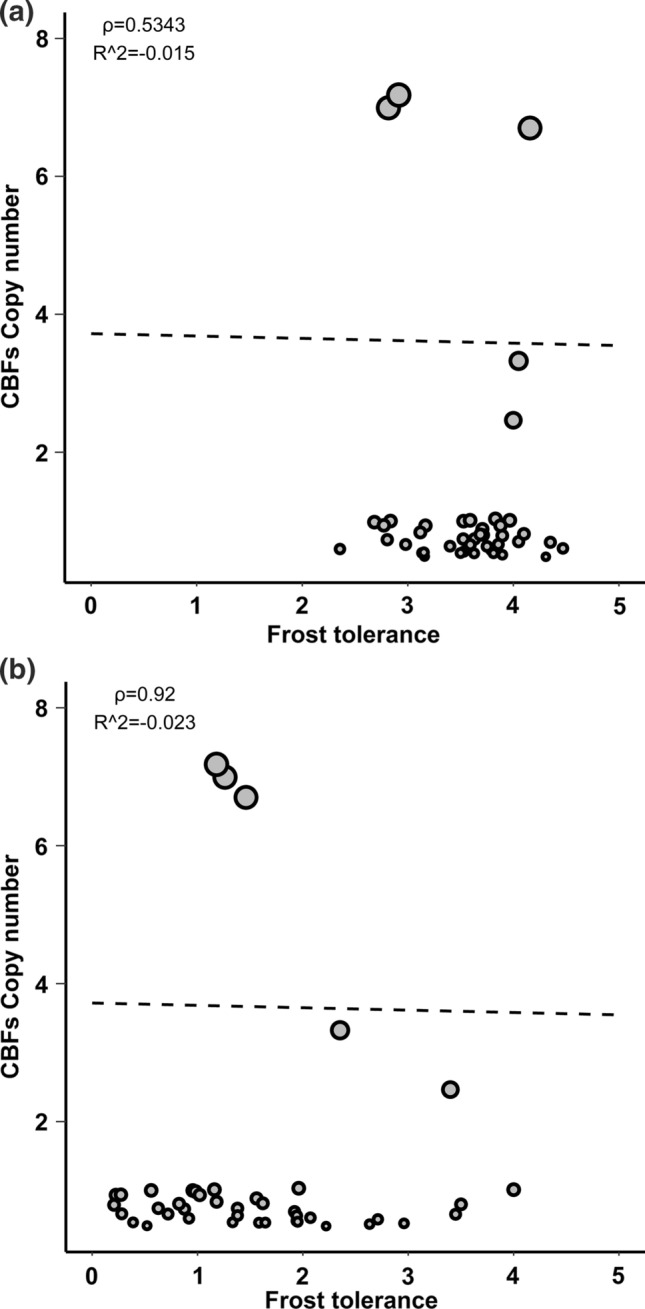
Linear regression models exclude a correlation between CNV of *CBF2a-CBF4b* and frost tolerance in our collection. Top panel reports on freezing test at − 6 °C, while bottom panel reports on − 9 °C test. Black lines represent the fitting values. Frost tolerance indicates the ability of a plant to growth back after frost treatment. A value of 0 indicates no growth back, while a value of 5 indicates s complete growth back and a well-developed plant producing tillers

To verify whether the allele mining using the WHEALBI panel was successful in detecting novel *CBF* alleles with a potential impact on frost tolerance, all sources of genetic diversity described above (CNV at *HvCBF2a-HvCBF4b*, *HvCBF14* haplotypes and *HvCBF2* paralogs) were combined and subjected to a freezing experiment in controlled conditions to test their level of frost tolerance. To normalize all tested samples for vernalization, only spring genotypes (i.e. accessions harbouring the *VRN1-1* or *VRN1-3* allele) were considered. Forty-five genotypes were carefully selected according to their haplotypes at each gene to evenly represent them in the sample (Supplementary Table S6). Unexpectedly, the Pearson’s correlation between frost tolerance and CNV was slightly negative and not significant (*r* =  − 0.099, *p* = 0.53 and *r* =  − 0.019, *p* = 0.9) at − 6 °C and − 9 °C, respectively (Supplementary Fig. S8a, b). A similar result was obtained when a multiple linear regression model with backward elimination was applied, that also included haplotypes at *VRN1*, *VRN2*, *HvCBF14* and *HvCBF2* paralogs as covariates (Fig. 8). Multiple linear regression with backward elimination retained only *HvCBF14* haplotypes as significant independent variable in the − 9 °C experiment and a combination of *VRNH1* alleles and *HvCBF14* haplotypes in the − 6° experiment.

**Fig. 9:**
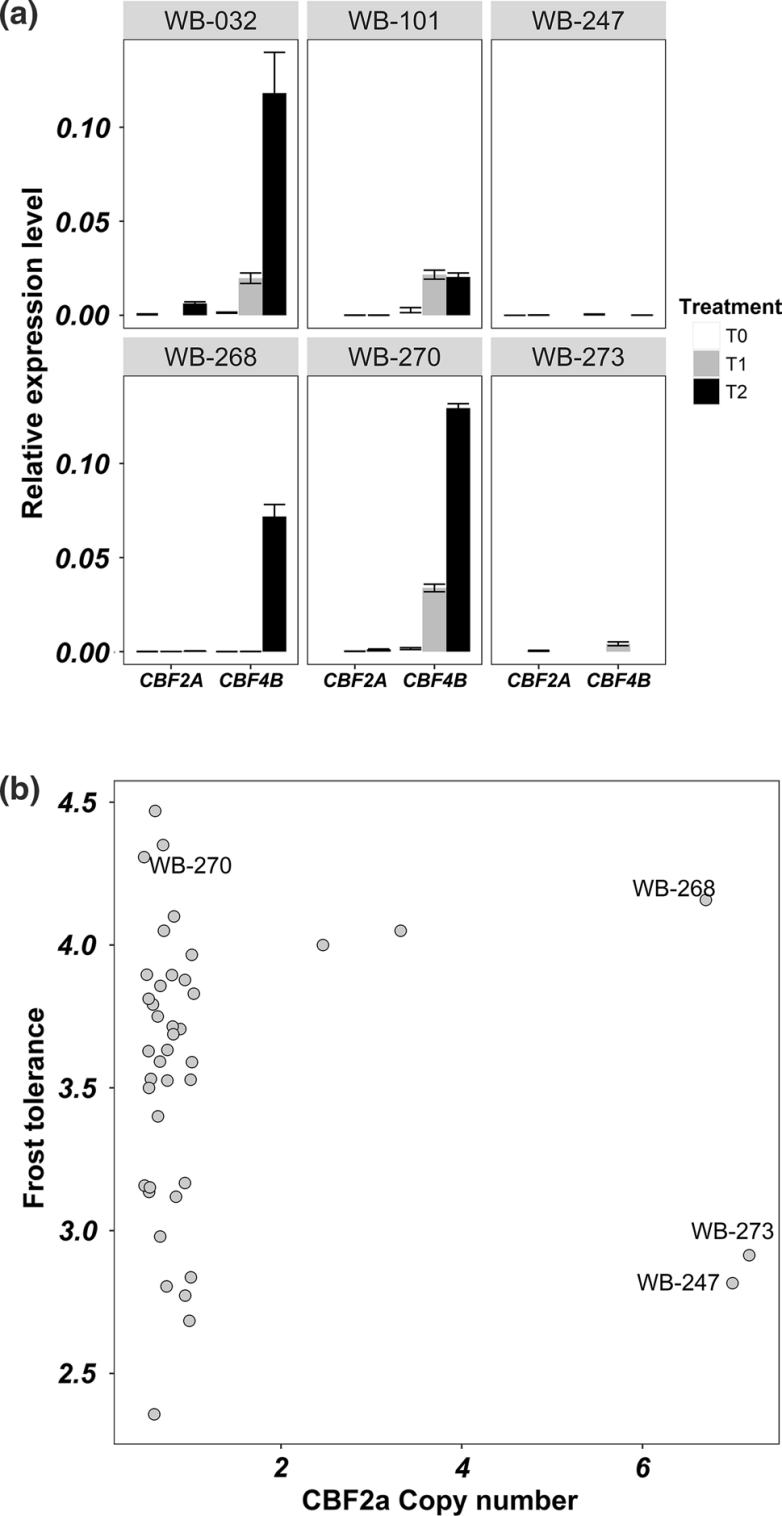
Top panel reports the expression profile of *CBF2a* and *CBF4b* in four representative barley genotypes and the two internal controls cvs. Morex (WB-101) and Pamina (WB-032); *T*0, *T*1 and *T*2 indicate cold treatment for 0,2 or 4 h. Bottom panel shows the distribution of genotypes according to their CNV and frost tolerance

**Fig. 10:**
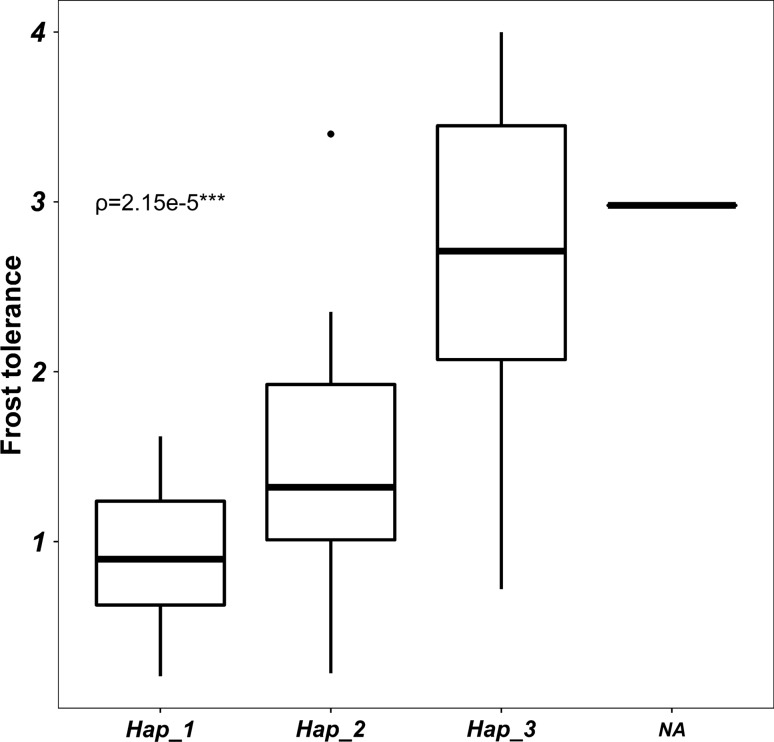
Box plot highlighting the positive effect of the haplotype 3 of *CBF14* on frost tolerance

Given that it is known, in both humans and plants, that CNV can affect the gene expression (Żmieńko et al. 2014; Geistlinger et al. 2018), we tested *HvCBF2a* and *HvCBF4b* gene expression by qRT-PCR in four genotypes showing a level of frost tolerance in disagreement to their CNV. WB-247 and 273 are high *CBFs* copy number genotypes but susceptible to frost. WB-270 is a low *CBFs* copy number genotype exhibiting a high level of frost resistance. Finally, WB-268 is a high *CBFs* copy number genotype and highly resistant to frost. qRT-PCR suggests that in our conditions frost tolerance is more related to *HvCBF4b* expression level rather than to CNV of *HvCBF2a-HvCBF4b* (Fig. 9a–b). Indeed, among the genotypes with the highest CNV, only WB-268 shows a high degree of frost tolerance and a high level of *HvCBF4b* expression. On the other side WB-270, which has a low CNV, shows a high degree of frost tolerance and a high level of *HvCBF4b* expression. Moreover, the latter shows a higher frost tolerance and *HvCBF4b* expression level than WB-268, supporting our hypothesis. Finally, when all genotypes in the experiment were analysed for frost tolerance according to haplotypes at *HvCBF14* a higher level of frost tolerance was observed for *HvCBF14* haplotype_3 as previously highlighted by multiple linear regression (Fig. 10).

We conclude that *CBF* mRNA accumulation, not *CBF* CN per se, controls the level of frost tolerance in barley and that variation in CN does not translate directly to variation in mRNA accumulation. Furthermore, our model highlights a highly significant positive correlation between frost tolerance and *HvCBF14* haplotypes (Fig. 10).

Finally, all the results of the multiple regression analyses were corroborated by analyses performed on independent frost tolerance tests performed with a subset of 40 of the 45 accessions mentioned above (Supplementary Data S2).

## Discussion

### Methods and tools for allele mining in barley

In the present study, exome data generated on the WHEALBI diverse barley collection (Bustos-Korts et al. 2019) have been used to develop and provide resources, tools, and methods to investigate allelic variation at multiple genes in barley*.* The resources have been trained by testing the diversity of the major regulators of frost tolerance in barley (Francia et al. 2004), namely *HvCBF2a*, *HvCBF4b, HvCBF14* and *Vrn-H1*. Previous investigations on small panels of accessions have provided evidence that *CBF* and *VRN* loci harbour a large genetic diversity in terms of SNP variations, deletions, paralogs and copy number variants, making this gene set a perfect proof of concept for testing the allele mining approach here described.

Genetic variation among individuals may occur in different forms beyond SNPs, collectively referred to as structural variations (Panchy et al. 2016). This kind of variation includes InDels, duplications, inversions and translocations of DNA segments ranging from few to thousand bases. So far, structural variation remains largely unexplored in plants, the lack of dedicated methods, resources and gold standard reference genomes collectively limit our ability to understand their contribution to phenotypes and population dynamics (Saxena et al. 2014; Zhou et al. 2019).

*Vrn-H1*, the major determinant of vernalization requirement in barley, is characterized by the presence of large InDels affecting its regulation and consequently the phenotype (von Zitzewitz et al. 2005; Hemming et al. 2009). The cultivar Morex, contributing the reference genome, is a spring genotype carrying a large deletion of 5154 bp in the first intron, thus hindering the identification of additional deletions or insertions. Replacing the spring allele in the Morex reference genomes by the winter allele of the cultivar Strider allowed the identification of 31 deletions, including all that are already known. These findings emphasize the importance of using the appropriate genome reference to maximize the amount of information that can be extracted from NGS data and point out to the usefulness of a pangenome reference.

CNVs are considered important players in driving the adaptation of different crop species to extreme conditions (Lye et al. 2009). Therefore, an increasing interest is turned to the identification and characterization of such variation in crops (Saxena et al. 2014). Here, we used an approach that relies on normalized reads count to evaluate CN at a single locus. As this approach aims to compare copy number variants for a single locus across multiple genotypes, it does not require a GC-bias correction as in other approaches (Benjamini et al. 2012). Using this method, we detected CNV for a 17 Kb segment on chromosome 5H harbouring two *CBF* genes, namely *HvCBF2a* and *HvCBF4b*. Next, we validated our in silico approach using digital PCR (Kanagal-Shamanna 2016) and the results confirmed the rank of each genotype tested with a correlation of 0.96 (*p*-value = 1.9*10^− 6^) between the WES and digital PCR data (Supplementary Fig. S6). Despite the high correlation, the CN value detected by digital PCR is lower than from WES and we hypothesize that WES data may be biased from the capture of a very close paralog that is not assembled in the reference genome. For instance, it is known that the cultivar Nure harbours at least two paralogs of *HvCBF4*, namely *HvCBF4a* and *HvCBF4b*, that are almost identical. Thus, while the assay for digital PCR has been designed in a way that can discriminate the paralogs, the WES cannot, and reads count increases consequently. Despite this limitation, our bioinformatic approach proved to be a simple, fast and robust way to investigate CNV at candidate genes.

Tandem or segmental duplications contribute to increase genetic and phenotypic variation giving rise to gene paralogs (Lynch and Conery 2000), a phenomenon that is very common in crop plants (Lye et al. 2019). Therefore, identifying and studying this kind of variation can help better understand plant phenotypes and adaptation to environment. However, the identification of gene paralogs is still a complicated task, especially in large and complex genomes (Djedatin et al. 2017). Here, we combined read count and analysis of heterozygous SNPs to identify gene paralogs. A similar approach has been previously used to discriminate gene paralogs in barley, Chinook salmon, mountain barberry and dusky parrotfish or to detect CNV in rice (Cantalapiedra et al., 2016, McKinney et al. 2016, Djedatin et al., 2017). Beyond combining read depth and heterozygous SNPs, we further corrected the data for the allele balance value by setting SNPs having a value lower than 0.25 as missing data. This approach allowed the identification of all know paralogs of *HvCBF2*, namely *HvCBF2a*, *HvCBF2b* and the *HvCBF2a/b* fusion and their combination in genotypes of the collection. These results support the usefulness of combining read count, read depth and allele balance to identify gene paralogs using WES data and provide new methods for genome-wide identification of gene paralogs which, in turn, will widen our knowledge on genomes evolution.

### New alleles regulating growth habit in barley

Overall, we tested the phenotypic effect of seven *VRN-H1* alleles and, when possible, each allele has been tested in different genetic backgrounds. Moreover, to test solely the effect of *VRN-H1,* all genotypes carried the wild allele of *VRN-H2*. The spring growth habit in barley is known to be determined by several InDels of variable size disrupting a regulatory region in the first intron of the gene; accordingly, we found one allele, named *VRN1-11*, characterized by a deletion of 8281 bp which determines the spring growth habit in four genetic backgrounds tested. Interestingly, one of these genotypes is insensitive to vernalization treatment but shows a delayed flowering compared to the others, and this may be due to allelic variation in other flowering genes like *ppd-H1* or *FT1*. By contrast, a second allele named *VRN1-15*, which carries a smaller deletion, shows a vernalization sensitive behaviour. Indeed, it can induce flowering only if at least a vernalization period of 2 weeks is provided, probably because a regulatory region is still retained. Three further alleles, *VRN1-12*, *VRN1-20*, and *VRN1-29* have been found to show a spring-type growth habit, but none of them carries large deletions. *VRN1-12* and *VRN1-20* are characterized by two short distinct deletions of 76 and 129 bp adjacent to the core region, but we exclude an effect on the phenotype given that they are present also in genotypes with a winter growth habit. The same conclusion can be drawn for *VRN1-29*, although in this case the deletion of 314 bp is in the promoter region. We then speculate that the spring phenotype may be due to some insertions in the regulatory region as in the case of the cultivar Varunda (Hemming et al. 2009). Finally, the alleles *VRN1-19* and *VRN1-26* are characterized by the same short deletions mentioned above and show a winter growth habit, with an increasing number of days to flower when vernalization is reduced. In conclusion, we identified at least one new deletion conferring spring growth habit in barley.

### Copy number variation of *HvCBF2a-HvCBF4b* segment is not associated with frost tolerance in spring barley

Copy number variation of the *HvCBF2a-HvCBF4b* segment in the *Fr-H2* locus has been known for a long time (Knox et al. 2010), and here, we explore it on a large barley collection. However, it is still debated which are the *HvCBFs* in the *Fr-H2* locus conferring frost tolerance. The 403 accessions used in the current study represent the largest germplasm collection tested so far for allelic diversity at the *Fr-H2* locus and it has been assembled to represent most of available genetic diversity of landraces and cultivated barley (Bustos-Kors et al. 2019). Indeed, the size and the composition of the collection allowed us to explore the effect of CNV on frost tolerance in a genetic background with unparalleled variability.

Recently, it has been observed that CNV of *HvCBF2a-HvCBF4b* is positively associated with frost tolerance in a panel of 41 winter, facultative and spring barley genotypes (Francia et al. 2016). In the present study, the georeferenced analysis in landraces and wild genotypes suggests a positive correlation between the copy number of *HvCBF2a-HvCBF4b* in a higher latitude with a colder climate; however, no positive association between copy number and frost tolerance has been found under controlled conditions. Likewise, the copy number of *HvCBF2a-HvCBF4b* is not associated with the expression level of both *CBFs* upon cold treatment, a result that agrees with Mareri et al. (2020), which, unlike this work, tested the expression of both *CBFs* in response to cold in the absence of light. Instead, at least in the genotypes tested, a correlation of the expression level of *HvCBF4b* with frost tolerance exists. Although these data might suggest a key role of *HvCBF4b* in controlling frost resistance, the effect of the combination of low temperatures and light cannot be excluded as previously suggested for this gene (Mareri et al. 2020). In addition, when the haplotypes of *HvCBF14* were considered as additional factor, a strong effect on frost tolerance was observed. Despite the absence of variants potentially affecting protein structure and function of *HvCBF14*, the correlation between its haplotypes and frost tolerance might be due to difference in the content of *cis elements* in the promoter region. This hypothesis is supported by the fact that the *HvCBF14* promoters of Morex and Nure, two cultivars with contrasting tolerance to cold, differ in the number of *cis elements* recognized by the transcription factor ICE1 (Mareri et al. 2020). Overall, these data suggest that low-temperature response might be primarily dependent on *HvCBF14* and that the CNV of *HvCBF2a-HvCBF4b* together with their expression might act as an additional level of regulation to fine-tune the response according to the latitude or the light quality/intensity.

The spring barley genotypes with increased frost tolerance described in this study (i.e. WB-270 and WB-268) might represent a resource for breeders to develop improved cultivars that anticipate the sowing and extend the plant cycle at high latitudes. Similarly, they may prove useful in South Europe where spring barleys are sown in autumn and need to overwinter, an option that may become more frequent due to milder winters. In this sense, another option is the exploitation of facultative barleys, but they have so far received less attention by breeders.

## Conclusion

The identification and exploitation of new allelic diversity is of key relevance to develop superior varieties and the development of resources, tools and methods to efficiently explore genetic diversity is of great relevance. Here, we present strategies and methods to study allelic variation in a large barley diversity panel going far beyond the analysis of SNPs and we suggest that CNV at the *Fr-H2* locus is not causative of an increased frost tolerance, but rather this may be attributable to a specific haplotype of *HvCBF14*. The WHEALBI collection together with the data protocols, methods generated in the present study represent a digital atlas of genome-wide allelic variants to analyse agronomic relevant traits.

## Supplementary Information

Below is the link to the electronic supplementary material.

Depiction of SNP-based haplotypes of VRN-H1, their alignment and geographic distribution. Geographic distribution was reported only for haplotypes with a frequency above 0.01. (TIF 2744 KB)

Depiction of SNP-based haplotypes of HvCBF2a, their alignment and geographic distribution. (TIF 1283 KB)

Multiple alignment of HvCBF2 paralogs. The polymorphic discriminating positions are in blue or black. (TIF 2591 KB)

Distribution of the raw read count at HvCBF2a in the collection. Raw number of reads mapping in the coding region of HvCBF2a is shown in ascending order from left to right. Yellow bars indicate genotype significantly differing from cv. Morex (TIFF 5156 KB)

Distribution of the raw read count at HvCBF4b in the collection. Raw number of reads mapping in the coding region of HvCBF4b is shown in ascending order from left to right. Yellow bars indicate genotype significantly differing from cv. Morex (TIFF 5156 KB)

Comparison of CNV values expressed as number of copies of the gene per haploid genome, revealed by whole genome exome sequencing (blue bars) and digital PCR (yellow bars) for 11 varieties from the WHEALBI panel representing the whole range of CN detected in HvCBF4b. In general, a nice correlation of WES and DPCR data can be observed (TIFF 5550 KB)

Copy number variation affecting the different paralogs of CBF2 in barley. The boxplot clearly shows how only HvCBF2a paralog (typical of cv. Morex) is affected by CNV while all the other are present as single copy. The paralog indicated as n2an2b although seems duplicated cannot be considered affected by CNV; indeed, it represents the contemporary presence of two different paralogs, HvCBF2a typical of cv. Nure and HvCBF2b which are present each as a single copy (TIFF 2269 KB)

Scatterplot reporting Pearson’s correlation between CN of HVCBF2a and genotype score for frost experiments at -6C° (top panel) and -9°C (bottom panel) (TIFF 519 KB)

Supplementary file9 (PDF 562 KB)

Supplementary file10 Genotypes included in the study and related information (XLSX 79 KB)

Supplementary file11 SNPs in the sequence of *VRN-H1* and relative effect as predicted by snpEff (XLSX 15 KB)

Supplementary file12 S3 Statistics on diversity of *VRN*-H1 in the collection (XLSX 10 KB)

Supplementary file13 Deletions in the sequence of VRN-H1 (XLSX 11 KB)

Supplementary file14 SNPs in the sequence of *HvCBF2a* and relative effect as predicted by snpEff (XLSX 10 KB)

Supplementary file15 Genotypes used for frost tolerance assay (XLSX 12 KB)

## Data Availability

The authors confirm that the data supporting the findings of this study are available within the article and its supplementary materials.
