# Supplementary figures and images for: Extensive allele mining discovers novel genetic diversity in the loci controlling frost tolerance in barley
Source: Theor Appl Genet. 2021 Nov 10;135(2):553–69. doi: 10.1007/s00122-021-03985-x (PMC8866391; doi:10.1007/s00122-021-03985-x)

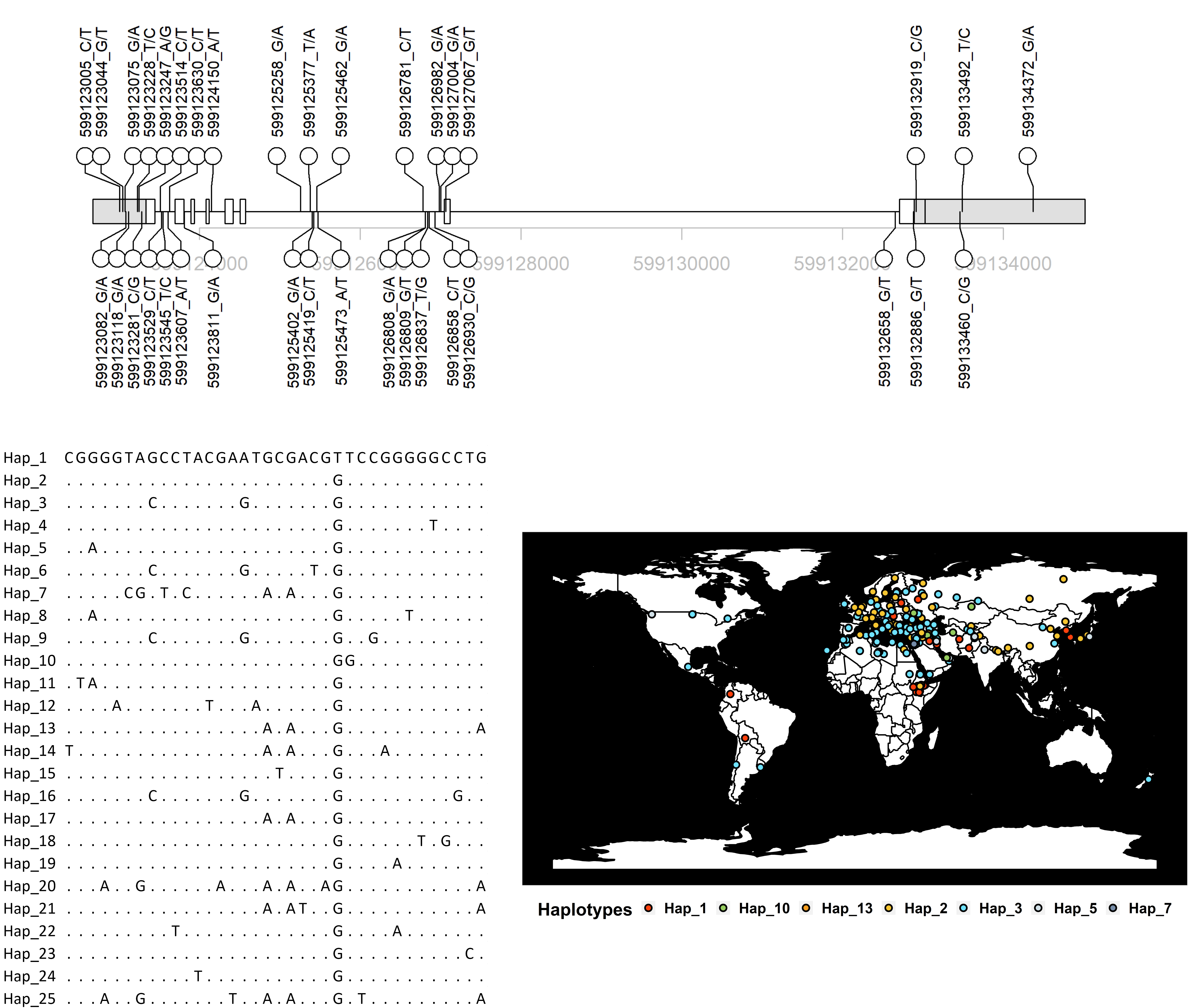

Supplement: Supplementary file 1 — Depiction of SNP-based haplotypes of VRN-H1, their alignment and geographic distribution. Geographic distribution was reported only for haplotypes with a frequency above 0.01. (TIF 2744 KB) [file 122_2021_3985_MOESM1_ESM.tif]

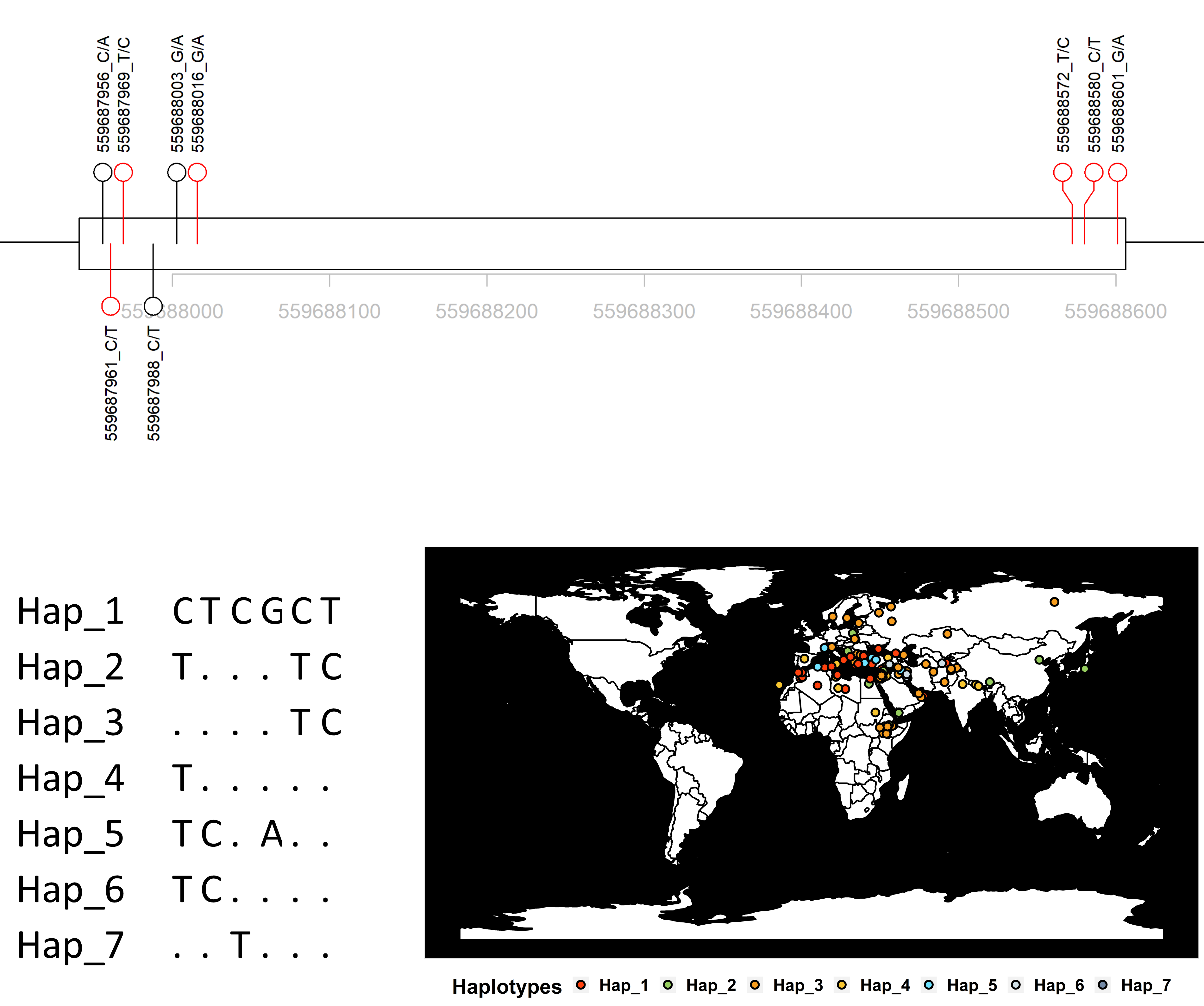

Supplement: Supplementary file 2 — Depiction of SNP-based haplotypes of HvCBF2a, their alignment and geographic distribution. (TIF 1283 KB) [file 122_2021_3985_MOESM2_ESM.tif]

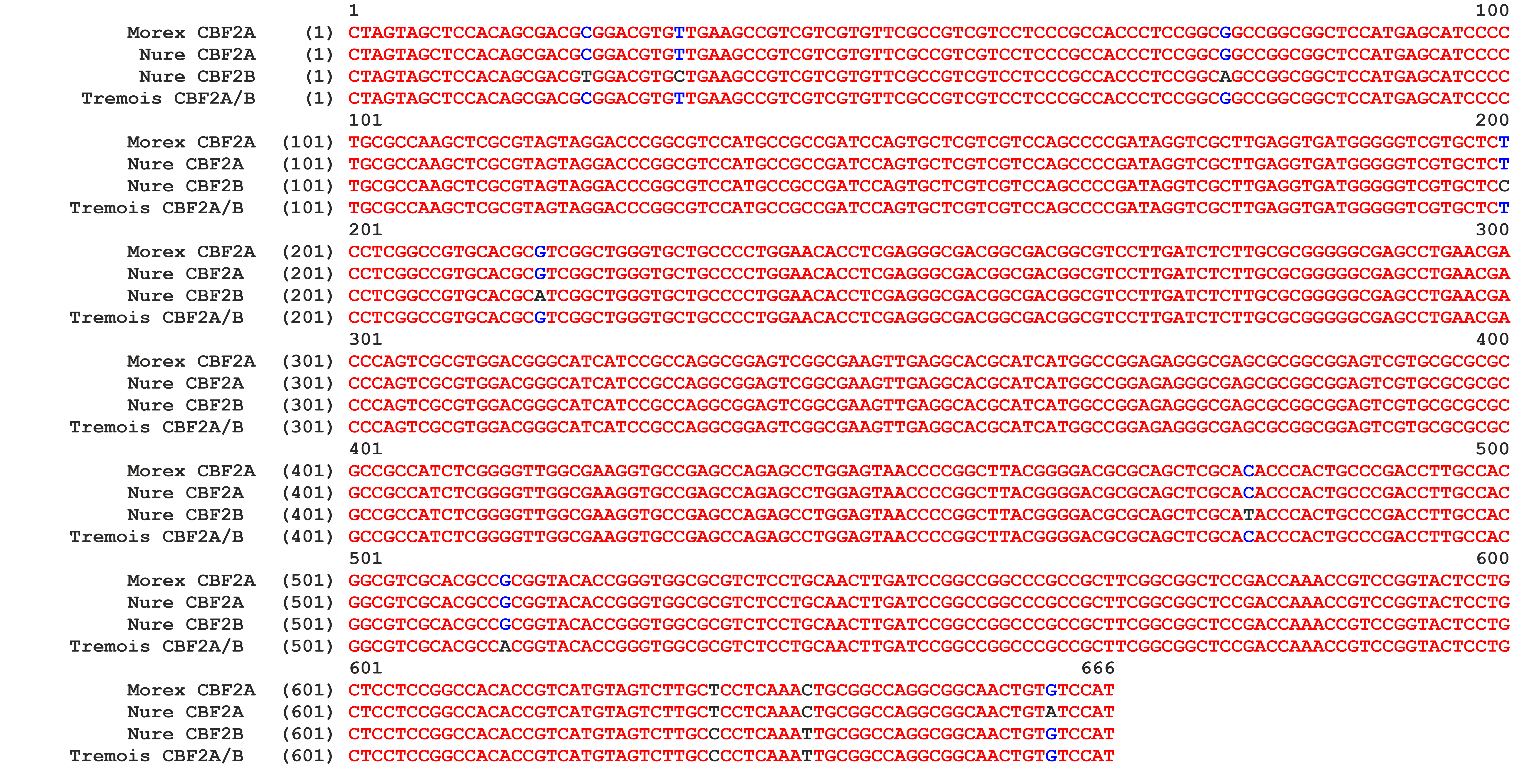

Supplement: Supplementary file 3 — Multiple alignment of HvCBF2 paralogs. The polymorphic discriminating positions are in blue or black. (TIF 2591 KB) [file 122_2021_3985_MOESM3_ESM.tif]

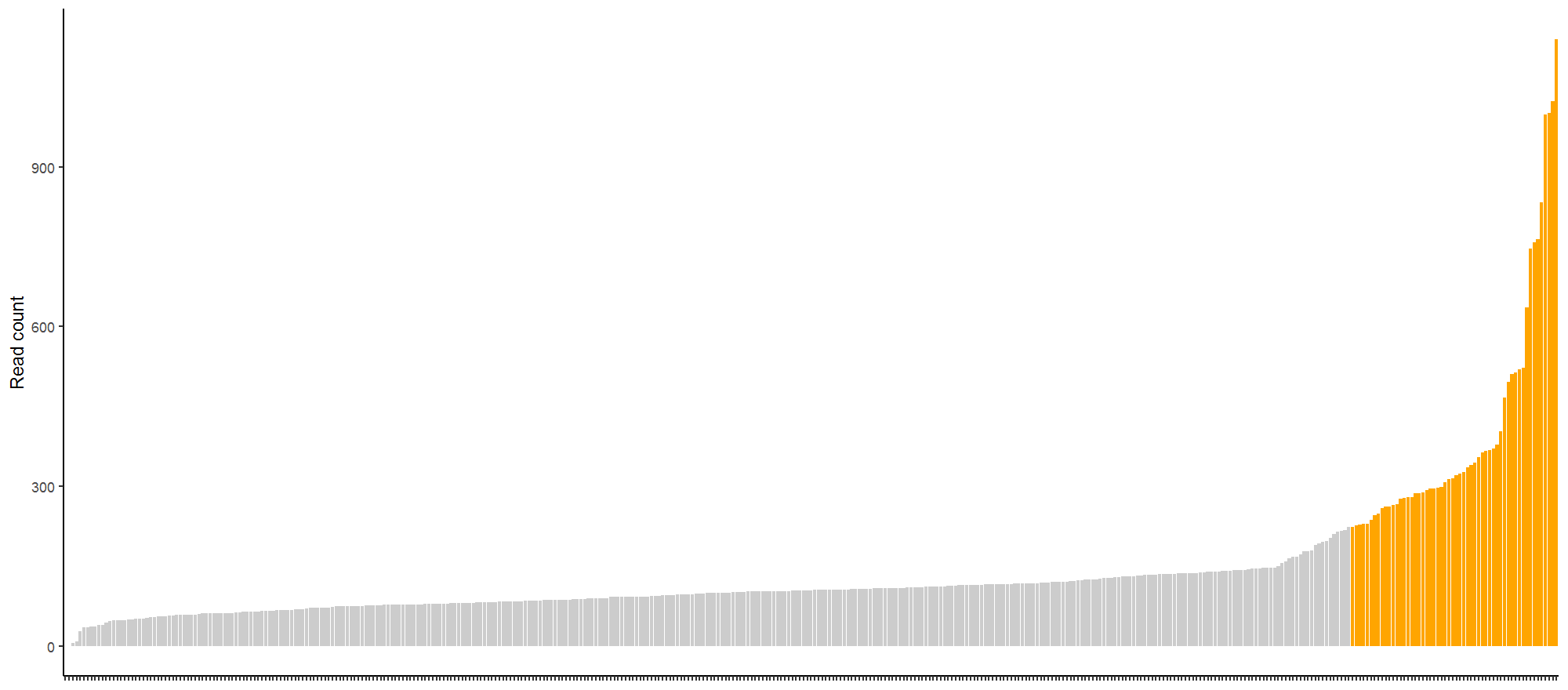

Supplement: Supplementary file 4 — Distribution of the raw read count at HvCBF2a in the collection. Raw number of reads mapping in the coding region of HvCBF2a is shown in ascending order from left to right. Yellow bars indicate genotype significantly differing from cv. Morex (TIFF 5156 KB) [file 122_2021_3985_MOESM4_ESM.tiff]

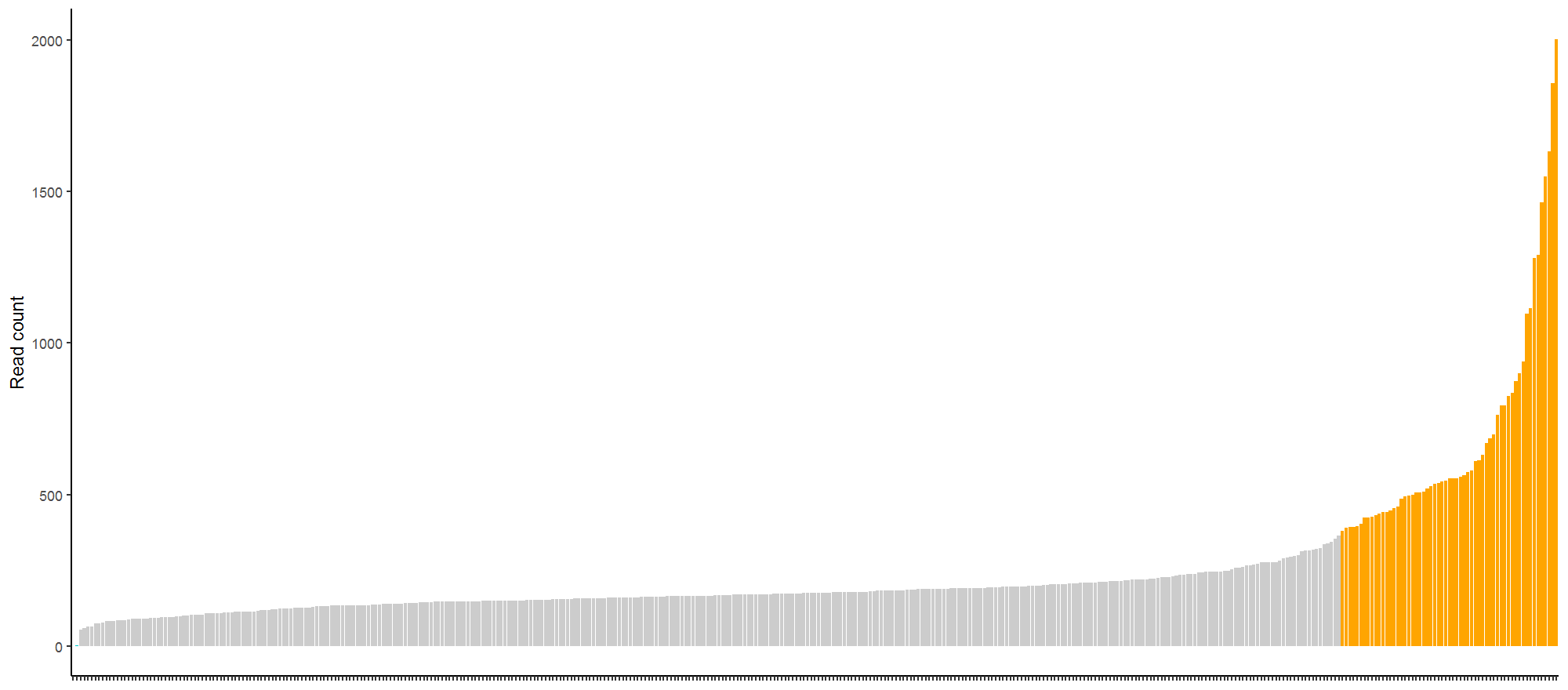

Supplement: Supplementary file 5 — Distribution of the raw read count at HvCBF4b in the collection. Raw number of reads mapping in the coding region of HvCBF4b is shown in ascending order from left to right. Yellow bars indicate genotype significantly differing from cv. Morex (TIFF 5156 KB) [file 122_2021_3985_MOESM5_ESM.tiff]

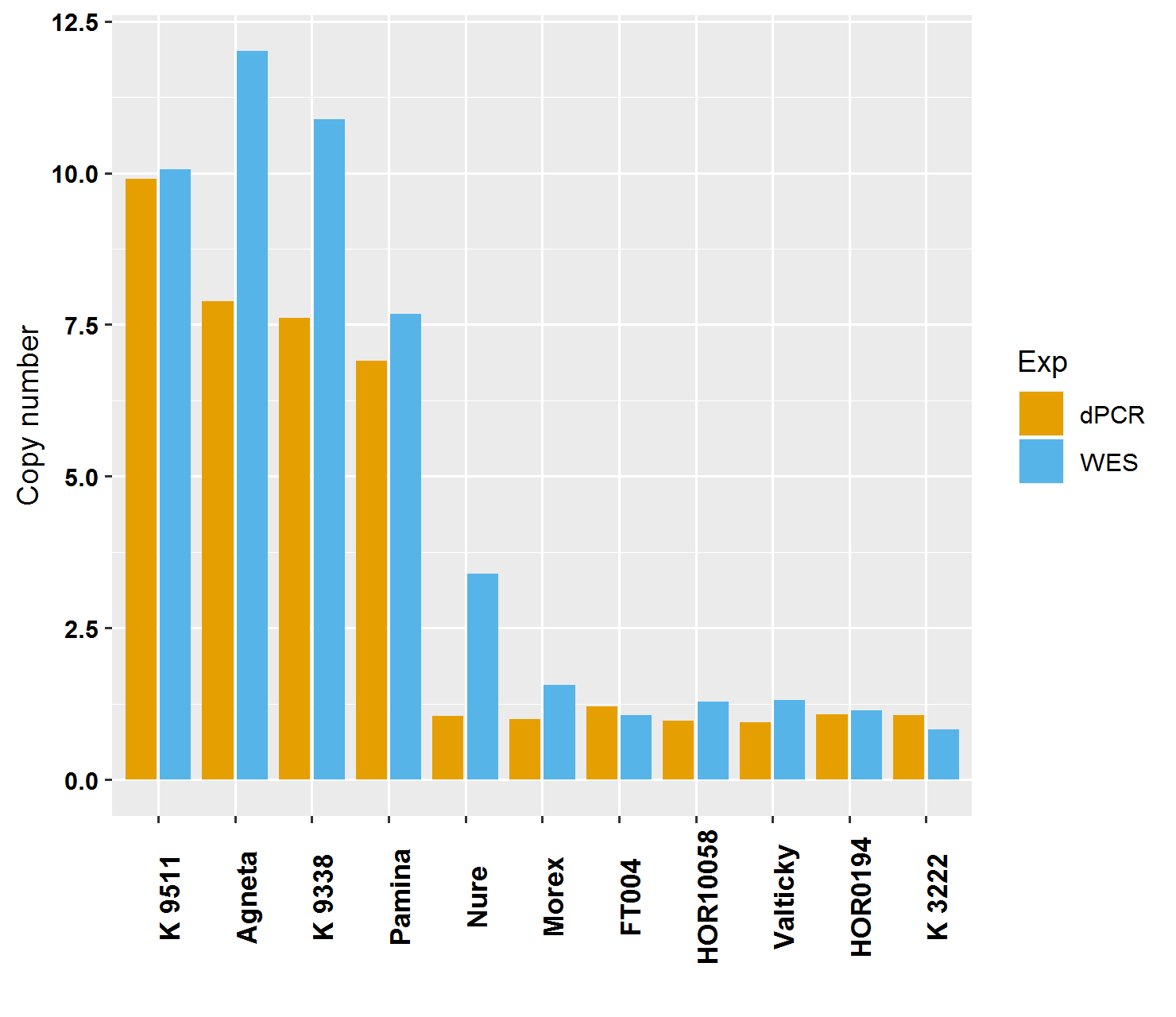

Supplement: Supplementary file 6 — Comparison of CNV values expressed as number of copies of the gene per haploid genome, revealed by whole genome exome sequencing (blue bars) and digital PCR (yellow bars) for 11 varieties from the WHEALBI panel representing the whole range of CN detected in HvCBF4b. In general, a nice correlation of WES and DPCR data can be observed (TIFF 5550 KB) [file 122_2021_3985_MOESM6_ESM.tiff]

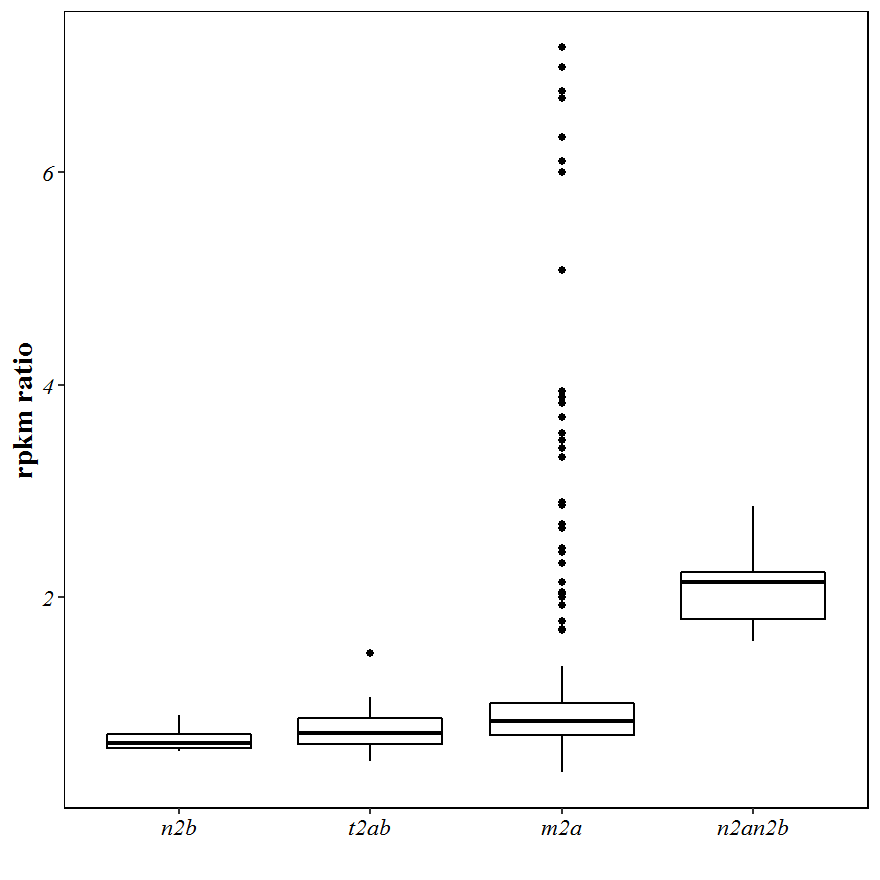

Supplement: Supplementary file 7 — Copy number variation affecting the different paralogs of CBF2 in barley. The boxplot clearly shows how only HvCBF2a paralog (typical of cv. Morex) is affected by CNV while all the other are present as single copy. The paralog indicated as n2an2b although seems duplicated cannot be considered affected by CNV; indeed, it represents the contemporary presence of two different paralogs, HvCBF2a typical of cv. Nure and HvCBF2b which are present each as a single copy (TIFF 2269 KB) [file 122_2021_3985_MOESM7_ESM.tiff]
