# Supplementary material for: Extensive allele mining discovers novel genetic diversity in the loci controlling frost tolerance in barley
Source: Theor Appl Genet. 2021 Nov 10;135(2):553–69. doi: 10.1007/s00122-021-03985-x (PMC8866391; doi:10.1007/s00122-021-03985-x)
Supplement: Supplementary file 9 — Supplementary file9 (PDF 562 KB) [file 122_2021_3985_MOESM9_ESM.pdf]

Data S1. Analysis of variance (ANOVA) and Tukey multiple comparisons of means were used to test the difference in days to head emergence between treatments (weeks of vernalization) in the experiment for vernalization requirement of different *VRN-H1* alleles. For each genotype, the ANOVA table, and the result of the Tukey multiple comparisons of means are reported below.

WB-363

```
            Df Sum Sq Mean Sq F value   Pr(>F)    
trt          3  667.7  222.58   26.09 3.35e-06 ***
Residuals   15  128.0    8.53                     
---
Signif. codes:  0 ‘***’ 0.001 ‘**’ 0.01 ‘*’ 0.05 ‘.’ 0.1 ‘ ’ 1
  Tukey multiple comparisons of means
    95% family-wise confidence level

Fit: aov(formula = dfgh ~ trt, data = i)

$trt
        diff        lwr         upr     p adj
W2-W0  -8.00 -13.323790 -2.67621033 0.0029702
W4-W0 -13.40 -18.723790 -8.07621033 0.0000152
W6-W0 -15.35 -20.996732 -9.70326834 0.0000060
W4-W2  -5.40 -10.723790 -0.07621033 0.0462656
W6-W2  -7.35 -12.996732 -1.70326834 0.0092806
W6-W4  -1.95  -7.596732  3.69673166 0.7543115
```

WB-078

```
            Df Sum Sq Mean Sq F value Pr(>F)
trt          3  843.1   281.0   1.913  0.181
Residuals   12 1762.7   146.9               
  Tukey multiple comparisons of means
    95% family-wise confidence level

Fit: aov(formula = dfgh ~ trt, data = i)

$trt
            diff       lwr       upr     p adj
W2-W0  -8.666667 -34.94450 17.611168 0.7636314
W4-W0 -18.666667 -44.94450  7.611168 0.2050591
W6-W0 -18.666667 -48.04618 10.712845 0.2838656
W4-W2 -10.000000 -32.75727 12.757272 0.5773807
W6-W2 -10.000000 -36.27783 16.277834 0.6790996
W6-W4   0.000000 -26.27783 26.277834 1.0000000
```

WB-364

```
            Df Sum Sq Mean Sq F value   Pr(>F)    
trt          3  386.0  128.68   13.47 0.000277 ***
Residuals   13  124.2    9.55                     
---
Signif. codes:  0 ‘***’ 0.001 ‘**’ 0.01 ‘*’ 0.05 ‘.’ 0.1 ‘ ’ 1
  Tukey multiple comparisons of means
    95% family-wise confidence level
```

```
Fit: aov(formula = dfgh ~ trt, data = i)

$trt
       diff        lwr       upr     p adj
W2-W0  -8.1 -14.185820 -2.014180 0.0085538
W4-W0 -11.5 -17.915018 -5.084982 0.0007754
W6-W0 -12.5 -18.915018 -6.084982 0.0003606
W4-W2  -3.4  -9.485820  2.685820 0.3918147
W6-W2  -4.4 -10.485820  1.685820 0.1971564
W6-W4  -1.0  -7.415018  5.415018 0.9669484
```

WB-101

```
            Df Sum Sq Mean Sq F value  Pr(>F)
trt          3  355.7  118.56   13.98 0.00032 ***
Residuals   12  101.7    8.48
---
Signif. codes:  0 '***' 0.001 '**' 0.01 '*' 0.05 '.' 0.1 ' ' 1
  Tukey multiple comparisons of means
    95% family-wise confidence level

Fit: aov(formula = dfgh ~ trt, data = i)

$trt
       diff        lwr        upr     p adj
W2-W0 -9.00 -15.113041 -2.8869588 0.0043595
W4-W0 -6.75 -12.863041 -0.6369588 0.0291925
W6-W0  2.50  -3.613041  8.6130412 0.6300740
W4-W2  2.25  -3.863041  8.3630412 0.7003805
W6-W2 11.50   5.386959 17.6130412 0.0005943
W6-W4  9.25   3.136959 15.3630412 0.0035456
```

WB-497

```
            Df Sum Sq Mean Sq F value Pr(>F)
trt          3  37263   12421    2736 <2e-16 ***
Residuals   14     64       5
---
Signif. codes:  0 '***' 0.001 '**' 0.01 '*' 0.05 '.' 0.1 ' ' 1
  Tukey multiple comparisons of means
    95% family-wise confidence level

Fit: aov(formula = dfgh ~ trt, data = i)

$trt
         diff         lwr           upr     p adj
W2-W0  -91.80  -95.954132  -87.64586752 0.0000000
W4-W0 -112.75 -117.128840 -108.37115989 0.0000000
W6-W0 -117.00 -121.154132 -112.84586752 0.0000000
W4-W2  -20.95  -25.104132  -16.79586752 0.0000000
W6-W2  -25.20  -29.116554  -21.28344634 0.0000000
W6-W4   -4.25   -8.404132   -0.09586752 0.0441994
```

WB-345

```
            Df Sum Sq Mean Sq F value   Pr(>F)
trt          3  24987    8329   101.8 2.79e-09 ***
Residuals   13   1064      82
```

```
---
Signif. codes:  0 '***' 0.001 '**' 0.01 '*' 0.05 '.' 0.1 ' ' 1
  Tukey multiple comparisons of means
    95% family-wise confidence level

Fit: aov(formula = dfgh ~ trt, data = i)

$trt
        diff        lwr        upr     p adj
W2-W0 -69.00  -88.38788 -49.612117 0.0000006
W4-W0 -88.25 -106.05891 -70.441092 0.0000000
W6-W0 -86.80 -103.59040 -70.009601 0.0000000
W4-W2 -19.25  -39.52633   1.026333 0.0650454
W6-W2 -17.80  -37.18788   1.587883 0.0763733
W6-W4   1.45  -16.35891  19.258908 0.9949555
```

WB-346

```
            Df Sum Sq Mean Sq F value Pr(>F)
trt          3  38404   12801    2139 <2e-16 ***
Residuals   14     84       6
---
Signif. codes:  0 '***' 0.001 '**' 0.01 '*' 0.05 '.' 0.1 ' ' 1
  Tukey multiple comparisons of means
    95% family-wise confidence level

Fit: aov(formula = dfgh ~ trt, data = i)

$trt
               diff         lwr        upr     p adj
W2-W0 -5.684342e-14   -4.770288   4.770288 1.0000000
W4-W0 -8.880000e+01  -93.297470 -84.302530 0.0000000
W6-W0 -9.650000e+01 -101.270288 -91.729712 0.0000000
W4-W2 -8.880000e+01  -93.570288 -84.029712 0.0000000
W6-W2 -9.650000e+01 -101.528325 -91.471675 0.0000000
W6-W4 -7.700000e+00  -12.470288  -2.929712 0.0017403
```

WB-Strider

```
            Df   Sum Sq   Mean Sq F value Pr(>F)
trt          3 9.69e-27 3.231e-27       1  0.418
Residuals   16 5.17e-26 3.231e-27
  Tukey multiple comparisons of means
    95% family-wise confidence level

Fit: aov(formula = dfgh ~ trt, data = i)

$trt
             diff           lwr          upr     p adj
W2-W0 0.000000e+00 -1.028563e-13 1.028563e-13 1.0000000
W4-W0 0.000000e+00 -1.028563e-13 1.028563e-13 1.0000000
W6-W0 2.842171e-14 -7.443463e-14 1.312780e-13 0.8576649
W4-W2 0.000000e+00 -1.028563e-13 1.028563e-13 1.0000000
W6-W2 2.842171e-14 -7.443463e-14 1.312780e-13 0.8576649
W6-W4 2.842171e-14 -7.443463e-14 1.312780e-13 0.8576649
```

WB-062

```
            Df Sum Sq Mean Sq F value   Pr(>F)    
trt          3 2669.0   889.7   178.8 1.63e-12 ***
Residuals   16   79.6     5.0                     
---
Signif. codes:  0 '***' 0.001 '**' 0.01 '*' 0.05 '.' 0.1 ' ' 1
  Tukey multiple comparisons of means
    95% family-wise confidence level

Fit: aov(formula = dfgh ~ trt, data = i)

$trt
       diff        lwr        upr     p adj
W2-W0 -12.6 -16.635965  -8.564035 0.0000007
W4-W0 -28.2 -32.235965 -24.164035 0.0000000
W6-W0 -27.0 -31.035965 -22.964035 0.0000000
W4-W2 -15.6 -19.635965 -11.564035 0.0000000
W6-W2 -14.4 -18.435965 -10.364035 0.0000001
W6-W4   1.2  -2.835965   5.235965 0.8295842
```

WB-352

```
            Df Sum Sq Mean Sq F value Pr(>F)  
trt          3  634.1  211.38   4.336 0.0252 *
Residuals   13  633.8   48.75                 
---
Signif. codes:  0 '***' 0.001 '**' 0.01 '*' 0.05 '.' 0.1 ' ' 1
  Tukey multiple comparisons of means
    95% family-wise confidence level

Fit: aov(formula = dfgh ~ trt, data = i)

$trt
        diff        lwr        upr     p adj
W2-W0 -16.75 -31.240923 -2.2590767 0.0218691
W4-W0 -11.00 -24.747297  2.7472969 0.1372775
W6-W0 -13.50 -27.990923  0.9909233 0.0712695
W4-W2   5.75  -7.997297 19.4972969 0.6212613
W6-W2   3.25 -11.240923 17.7409233 0.9107540
W6-W4  -2.50 -16.247297 11.2472969 0.9492516
```

WB-353

```
            Df Sum Sq Mean Sq F value   Pr(>F)    
trt          3  31628   10543   186.2 6.28e-11 ***
Residuals   13    736      57                     
---
Signif. codes:  0 '***' 0.001 '**' 0.01 '*' 0.05 '.' 0.1 ' ' 1
  Tukey multiple comparisons of means
    95% family-wise confidence level

Fit: aov(formula = dfgh ~ trt, data = i)

$trt
               diff        lwr         upr     p adj
W2-W0 -2.842171e-14  -18.47736  18.4773633 1.0000000
W4-W0 -7.960000e+01  -93.56757 -65.6324263 0.0000000
W6-W0 -9.420000e+01 -108.16757 -80.2324263 0.0000000
W4-W2 -7.960000e+01  -98.07736 -61.1226367 0.0000001
```

```
W6-W2 -9.420000e+01 -112.67736 -75.7226367 0.0000000
W6-W4 -1.460000e+01  -28.56757  -0.6324263 0.0394067
```

WB-135

```
            Df Sum Sq Mean Sq F value  Pr(>F)   
trt          3 171.23   57.08   10.61 0.00141 **
Residuals   11  59.17    5.38                   
---
Signif. codes:  0 '***' 0.001 '**' 0.01 '*' 0.05 '.' 0.1 ' ' 1
  Tukey multiple comparisons of means
    95% family-wise confidence level

Fit: aov(formula = dfgh ~ trt, data = i)

$trt
           diff        lwr       upr     p adj
W2-W0 -6.833333 -12.164250 -1.502417 0.0121306
W4-W0 -9.083333 -14.414250 -3.752417 0.0015910
W6-W0 -8.583333 -13.914250 -3.252417 0.0024608
W4-W2 -2.250000  -7.185469  2.685469 0.5403381
W6-W2 -1.750000  -6.685469  3.185469 0.7154285
W6-W4  0.500000  -4.435469  5.435469 0.9896113
```

WB-137

```
            Df Sum Sq Mean Sq F value  Pr(>F)   
trt          3 1204.4   401.5   9.464 0.00113 **
Residuals   14  593.9    42.4                   
---
Signif. codes:  0 '***' 0.001 '**' 0.01 '*' 0.05 '.' 0.1 ' ' 1
  Tukey multiple comparisons of means
    95% family-wise confidence level

Fit: aov(formula = dfgh ~ trt, data = i)

$trt
            diff        lwr       upr     p adj
W2-W0 -19.000000 -30.972664 -7.027336 0.0020152
W4-W0 -10.266667 -24.091508  3.558174 0.1828741
W6-W0 -18.800000 -30.772664 -6.827336 0.0022057
W4-W2   8.733333  -5.091508 22.558174 0.2981708
W6-W2   0.200000 -11.772664 12.172664 0.9999568
W6-W4  -8.533333 -22.358174  5.291508 0.3164087
```

WB-401

```
            Df Sum Sq Mean Sq F value Pr(>F)
trt          3  137.8   45.92   0.742  0.545
Residuals   14  866.7   61.91               
  Tukey multiple comparisons of means
    95% family-wise confidence level

Fit: aov(formula = dfgh ~ trt, data = i)

$trt
       diff        lwr      upr     p adj
W2-W0 -3.80 -19.141559 11.54156 0.8875258
```

```
W4-W0 -3.75 -19.921423 12.42142 0.9051853
W6-W0  2.60 -12.741559 17.94156 0.9594569
W4-W2  0.05 -15.291559 15.39156 0.9999997
W6-W2  6.40  -8.064160 20.86416 0.5859487
W6-W4  6.35  -8.991559 21.69156 0.6349734
```

WB-427

```
            Df Sum Sq Mean Sq F value Pr(>F)    
trt          3  992.1   330.7   87.54  2e-08 ***
Residuals   12   45.3     3.8                   
---
Signif. codes:  0 '***' 0.001 '**' 0.01 '*' 0.05 '.' 0.1 ' ' 1
  Tukey multiple comparisons of means
    95% family-wise confidence level

Fit: aov(formula = dfgh ~ trt, data = i)

$trt
            diff        lwr         upr     p adj
W2-W0  -1.066667  -5.280849   3.1475155 0.8743253
W4-W0 -13.000000 -17.711599  -8.2884011 0.0000153
W6-W0 -17.866667 -22.080849 -13.6524845 0.0000001
W4-W2 -11.933333 -16.147515  -7.7191512 0.0000117
W6-W2 -16.800000 -20.449589 -13.1504112 0.0000001
W6-W4  -4.866667  -9.080849  -0.6524845 0.0224354
```

Data S2. Results of FT (frost tolerance)-tests performed on a larger subset of WHEALBI accessions were extracted for 40 accessions in common with the set of accessions studied in the current work. The FT-tests were done on leaves cut from plants grown in the field (see Di Gennaro et al. 2018 for more information on the experiment) and subsequently subjected to a controlled temperature freezing test (method described and tested in Badeck & Rizza, 2015). Thus, these samples were representative for plants cold acclimated under outdoor winter conditions, sampled on November 25th 2015 (F1), December 11th 2015 (F2), January 19th 2016 (F3), as well as at January 16th 2017 (F4) from a repetition of the field experiment. In addition, the same accessions had been grown under controlled conditions in a growth cabinet and cold acclimated for four weeks at either optimal hardening condition of 1°C / 3°C night/day temperatures (G1, G2) or at suboptimal conditions at 7°C / 12 °C night/day temperatures (G3, G4). Minimum temperature during the freezing tests were -14 °C for the field sampled leaves, -13 °C for optimal and -8 °C for suboptimal hardening conditions.
The maximum yield of photosystem II (Fv/Fm) after 24 h of recovery was used as an indicator of leaf damage. Multiple linear regression of Fv/Fm on *VRN-H1*, *VRN-H2* alleles, *HvCBF14* haplotypes and copy number variation of *HvCBF2a* and *HvCBF4b* with backward elimination was used to study significant effects of the genes on FT.

Data S2: results of multiple linear regression of Fv/Fm on *VRN-H1*, *VRN-H2* alleles, *HvCBF14* haplotypes and copy number variation of *HvCBF2a* and *HvCBF4b* within the eight FT experiments. Results for regression on accession means and in parentheses for the full set of individual measurements. In the column “Genes retained in the model” only if the genes retained differ between the model based on accession means and full set the genes for the latter are given in parentheses. Genes in bold had significant effects at p< 0.05. Non-significant gene effects (p > 0.05) that were nevertheless retained in the model are shown in plain text.

Experiment	Genes retained in the model	Slope of CNV	R2
F1	VRNH1, VRNH2		0.49 (0.23)
F2	CBF14, CNV CBF4b	0.007 (0.007)	0.31 (0.14)
F3	CBF14, VRNH2, CNV CBF4b	- 0.011 (-0.011)	0,70 (0.55)
F4	CNV CBF4b (VRNH2, CNV CBF4b)	-0.017 (-0.018)	0.08 (0.07)
G1	VRNH1, VRNH2		0.22 (0.09)
G2	VRNH1, CBF14, CNV CBF4b	-0.034 (-0.036)	0.54 (0.33)
G3	VRNH1, CBF14, VRNH2, CNV CBF2a, CBF4b	0.302 -0.205 (0.299, -0.111)	0.56 (0.44)
G4	CBF14		0.13 (0.09)

As was the case in the survival test, the differences in FT between spring type accessions as well as the fraction of variability explained by gene effects were small. In tests F1, F2, G1 and G4 most leaves were strongly damaged and thus the tests had a low discriminative capacity. The vernalization genes and CBF14 haplotypes had the most pronounced effects on FT across all experiments. CNV of CBF2 and CBF4 did not exert significant effects in the experiments on plants grown under controlled conditions. In experiment G3 the highly correlated CNV of CBF2a and CBF4b (see main text) had non-significant opposite effects, indicating that these results probably trace some hidden effects in the model that are not due to CNV of these two CBF. In the experiments with leaves sampled in the field CNV of CBF4b was associated with FT with opposite sign in F2 versus F3 and F4. Overall, higher CNV of CBF4b tended to be associated with lower FT.

References

Badeck F.-W. & Rizza F. (2015) A Combined Field/Laboratory Method for Assessment of Frost Tolerance with Freezing Tests and Chlorophyll Fluorescence. Agronomy, 5, 71-88.

Di Gennaro S.F., Rizza F., Badeck F.W., Berton A., Delbono S., Gioli B., Toscano P., Zaldei A. & Matese A. (2018) UAV-based high-throughput phenotyping to discriminate barley vigour with visible and near-infrared vegetation indices. International Journal of Remote Sensing, 39, 5330-5344.
